# Evaluating the summer landscapes of predation risk and forage quality for elk (*Cervus canadensis*)

**DOI:** 10.1002/ece3.9201

**Published:** 2022-08-11

**Authors:** John Terrill Paterson, Kelly M. Proffitt, Nicholas J. DeCesare, Justin A. Gude, Mark Hebblewhite

**Affiliations:** ^1^ Department of Ecology Montana State University Bozeman Montana USA; ^2^ Montana Fish Wildlife and Parks Bozeman Montana USA; ^3^ Department of Ecosystem and Conservation Sciences University of Montana Missoula Montana USA

**Keywords:** *Canis lupus*, *Cervus canadensis*, non‐consumptive effects, predation risk, *Puma concolor*, risk effects

## Abstract

The recovery of carnivore populations in North American has consequences for trophic interactions and population dynamics of prey. In addition to direct effects on prey populations through killing, predators can influence prey behavior by imposing the risk of predation. The mechanisms through which patterns of space use by predators are linked to behavioral response by prey and nonconsumptive effects on prey population dynamics are poorly understood. Our goal was to characterize population‐ and individual‐level patterns of resource selection by elk (*Cervus canadensis*) in response to risk of wolves (*Canis lupus*) and mountain lions (*Puma concolor*) and evaluate potential nonconsumptive effects of these behavioral patterns. We tested the hypothesis that individual elk risk‐avoidance behavior during summer would result in exposure to lower‐quality forage and reduced body fat and pregnancy rates. First, we evaluated individuals' second‐order and third‐order resource selection with a used‐available sampling design. At the population level, we found evidence for a positive relationship between second‐ and third‐order selection and forage, and an interaction between forage quality and mountain lion risk such that the relative probability of use at low mountain lion risk increased with forage quality but decreased at high risk at both orders of selection. We found no evidence of a population‐level trade‐off between forage quality and wolf risk. However, we found substantial among‐individual heterogeneity in resource selection patterns such that population‐level patterns were potentially misleading. We found no evidence that the diversity of individual resource selection patterns varied predictably with available resources, or that patterns of individual risk‐related resource selection translated into biologically meaningful changes in body fat or pregnancy rates. Our work highlights the importance of evaluating individual responses to predation risk and predator hunting technique when assessing responses to predators and suggests nonconsumptive effects are not operating at a population scale in this system.

## Graphical abstract

Example of predicted relative probabilities of selection (3rd order) for a small sample of individual female elk (*n* = 9), based on the underlying landscapes of risk and forage quality. The lower panel illustrates the variety of predicted relative probabilities of selection (3rd order) for elk in response to the underlying landscape of forage quality and risk. To aid illustration, all values were binned into 10 quantiles and coded from low (blue) to high (red).

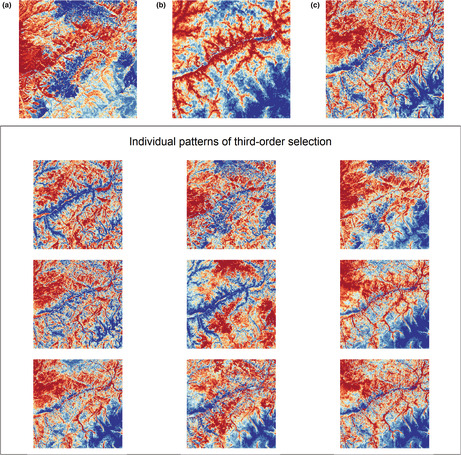

## 1 INTRODUCTION

The recovery of large carnivores in North America is an important ecological change that has the potential to impact trophic interactions at multiple scales through various effects on prey species (Ripple et al., 2014). In addition to the direct effects on prey populations from killing and consumption, predators also have the potential to influence the behavioral patterns of prey with the threat of predation (Lima, 1998, 2002; Lima & Dill, 1990). The perception of predation risk should induce behavioral strategies in prey seeking to balance access to high‐quality forage with avoidance of predation, such that patterns of resource use by prey are impacted by those of predators (Brown, 1999; Sih, 1984; Verdolin, 2006). However, the pathways through which the space use patterns of predators are subsequently perceived as risk by prey are not well understood (Gaynor et al., 2019; Schmidt & Kuijper, 2015), a situation at least partly complicated by the diverse meanings of the term “risk” in empirical evaluations of predator–prey interactions (Moll et al., 2017). The probability of predation (i.e., being killed) depends on multiple processes (e.g., time spent vulnerable to encountering a predator, the probability of encountering a predator, and the probability of death conditional on an encounter [Lima & Dill, 1990]), and prey responses to the probability of predation are likely a complex interaction between the style of predation, scale, and landscape structure. For example, there is some evidence to suggest that elk (*Cervus canadensis*) shift their large‐scale distribution from structurally simple landscapes to structurally complex landscapes in response to a cursorial predator (wolves [*Canis lupus*]; Creel & Winnie, 2005; Creel et al., 2005), yet other evidence suggests that predation from coursing predators is not connected to specific structural features, and habitat‐specific signals of predation by coursers are weak (Kauffman et al., 2010; Schmidt & Kuijper, 2015). Avoiding predation risk may therefore take the form of temporal avoidance whereby elk change their patterns of resource use only when wolves are present (Creel et al., 2005; Cusack et al., 2020). In contrast, ambush or stalking predators (e.g., mountain lions [*Puma concolor*]) that rely on fine‐scale landscape features (e.g., stalking cover) to approach prey should generate spatially predictable cues, with the net result that habitat signals of predation should be much stronger (Laundré & Hernández, 2003; Podgórski et al., 2008).

Underlying much of the empirical evaluation of the response of prey to the landscape of risk posed by predators is the tacit assumption that estimated population‐level responses of prey to the perception of risk are a representative marginalization of individual‐level responses (Gaynor et al., 2019; Pettorelli et al., 2015). Deviations from the expected relationship between the resource use patterns of prey and the spatial arrangement of predators can arise from a multitude of factors related to individual characteristics (Gaynor et al., 2019). Given that the response of prey to the risk of predation is predicated on a fitness‐related trade‐off between foraging opportunities and predation, it is unsurprising that balance would vary among individuals of different ages, physiological conditions, levels of exposure to risk, individuals subject to different ecological constraints on the ability to respond (Clark, 1994; Gaynor et al., 2019; Schmidt & Kuijper, 2015; Valeix et al., 2009), or differences attributable to components of prey personality (i.e., repeatable characteristics across time and context) such as the position along a bold‐shy continuum (McArthur et al., 2014; Sloan Wilson et al., 1994). Although there are few assessments of among‐individual heterogeneity in response to predation risk (Gaynor et al., 2019; Pettorelli et al., 2015), a growing body of evidence suggests that individual variation in the perception of (or response to) risk has implications for population‐level processes (Abbey‐Lee & Dingemanse, 2019; Abbey‐Lee et al., 2016; Mumma et al., 2017).

The integrated result of individual trade‐offs associated with behavioral responses to the landscape of risk from predators has the potential to impact the population dynamics of prey (nonconsumptive effects, or NCEs). While often overlooked, NCEs may have important demographic effects on prey populations when predator‐induced alteration of prey behavior results in changes to reproduction or survival (Lima, 1998; Schmitz et al., 2004). Evidence of NCEs on prey populations has been shown in small‐scale experimental studies (Schmitz et al., 1997); however, the presence and extent of NCEs in large carnivore predator–prey systems has been debated (Creel et al., 2011; Middleton et al., 2013a; White et al., 2011). There are two primary mechanisms by which predation risk can result in NCEs on prey population dynamics: the predation stress hypothesis and the predator‐sensitive food hypothesis (Boonstra et al., 1998; Creel et al., 2009). Although the predation stress hypothesis (risk induces elevated levels of stress hormones with deleterious consequences to survival and reproduction) has not been supported in elk‐wolf systems, the predator‐sensitive food hypothesis (risk‐related behavioral changes in foraging patterns result in nutritional costs that limit reproduction) has received some support (Christianson & Creel, 2010; Creel et al., 2009, 2011; Sinclair & Arcese, 1995; White et al., 2011). To our knowledge, the presence of NCEs in elk‐lion systems has not been addressed. Potential risk‐related variation in foraging patterns is particularly relevant for understanding the population dynamics of large ungulates, for whom the quality (i.e., digestible energy) and not quantity (biomass) of forage during the summer months has been linked to population vital rates (Cook et al., 2013; McArt et al., 2009; White, 1983). The presence of NCEs and the magnitude on their impact on ungulate population dynamics has implications for management decisions, that is, identifying the best management strategy would require understanding how a risk‐averse foraging strategy translated into variation in nutritional condition and vital rates, and if such variation affected population dynamics (Sheriff et al., 2020).

The predator‐sensitive food hypothesis requires two logical requisites: (1) a correlation between forage quality and predation risk such that predator‐sensitive foraging results in a reduction of access to high‐quality forage, and (2) the trade‐off impacts physiological parameters related to vital rates underlying population dynamics (e.g., survival and reproduction). For ungulates in particular, there have been many recent studies of the effects of predation risk on large herbivore spatial ecology, survival, and reproduction, as well as the importance of nutrition to ungulate reproductive performance (Cook et al., 2004; Middleton et al., 2013b; Parker et al., 2009). In combination, these findings suggest the possibility that risk‐related behavioral changes in habitat use or risk‐sensitive foraging could result in reduced nutritional condition and reproductive performance. To date, evaluations of NCEs in elk‐wolf systems have primarily focused on winter effects. Creel et al. (2009) investigated NCEs in a GYE elk‐wolf system and concluded that behavioral avoidance of wolf risk led to changes in elk foraging patterns during winter that resulted in nutritional limitations on pregnancy. However, Middleton et al. (2013a) investigated NCEs in a different GYE elk‐wolf system and found no evidence that risk of predation during winter was associated with reduced elk body fat or pregnancy. Although we are aware of no assessment of NCEs in an elk‐lion system, recent work has suggested a behavioral avoidance response of elk to mountain lions in an elk‐wolf‐lion system that may result in NCEs if there is a trade‐off in access to high‐quality forage as a consequence (Kohl et al., 2019). Acquisition of high‐quality nutritional resources during late summer is a well‐documented, important driver of the level of body fat that elk accrue by the fall breeding period (Cook et al., 2013, 2016; Monteith et al., 2013; Proffitt et al., 2016), the subsequent probability of pregnancy (Cook et al., 2013), carry‐over effects on late‐winter body fat (Cook et al., 2013; Middleton et al., 2013b), and even birthweight and survival of neonates in the following summer (Griffin et al., 2011). Thus, if risk‐avoidance behaviors result in nutrition‐mediated demographic consequences, the behaviors should at least partially occur during the summer period and be measurable as changes in elk habitat use, fall body fat accrual, and fall pregnancy status. To date, changes in summer elk forage selection induced by risk‐avoidance of multiple predators and subsequent fall body fat and pregnancy rates, the likely mechanisms which could lead to risk‐induced demographic effects, have not been documented.

Using a multispecies carnivore‐elk system in west‐central Montana, our goals were to: (1) evaluate how spatial use patterns of elk reflect the underlying landscapes of nutrition and predation risk from wolves and mountain lions, and (2) evaluate the evidence for potential NCEs in this system. First, we investigated the intrinsic response of individuals to forage and predation risk at multiple scales. At both the broad scale of the entire population summer range and the finer scale within individual summer home ranges, we tested for effects of predation risk on selection of summer‐range forage quality using mixed‐effects models incorporating individual variation in responses. Second, we assessed the potential for demographic consequences of trade‐offs between risk and forage quality by evaluating the strength of evidence for an association between the magnitude of the trade‐off and the fall body fat and probability of pregnancy for individual elk, two key physiological parameters related to ungulate population dynamics.

## 2 METHODS

### 2.1 Study area

**FIGURE 1:**
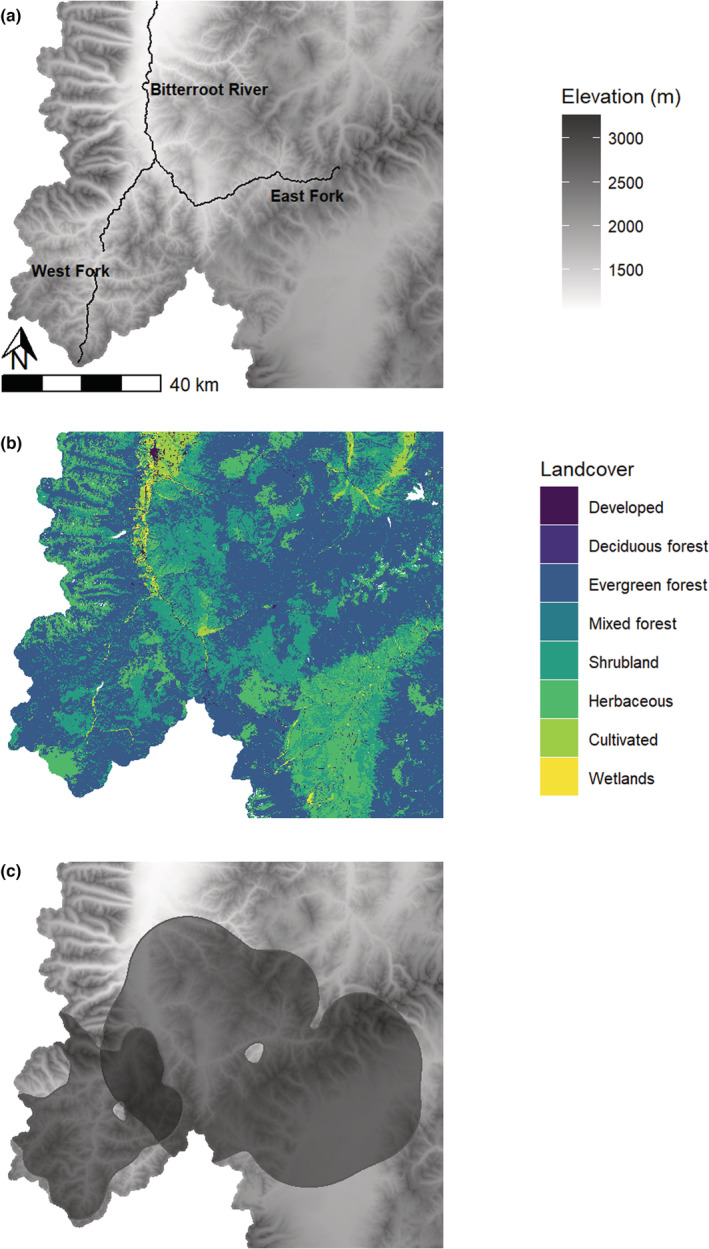
The study area was located in west‐Central Montana along the bitterroot river, including its east and west forks, and had high variation in elevation (panel a). Landcover type was dominated by forest and shrublands (panel b), based on the 2016 National Landcover Database classification scheme. Spatial locations, body fat and pregnancy information came from two populations (east fork and west fork) populations in the study area (panel c, showing the 99% contour of the utilization distribution estimated from GPS locations).

The 3350 km^2^ study area is located in the southern Bitterroot Valley in west‐central Montana. The area encompasses the headwaters of the West Fork and East Fork of the Bitterroot River (Figure 1). The West Fork area consists of rugged terrain, with elevations ranging from 1200 m in the valley bottom to 3300 m along the Bitterroot crest. Most of the area is heavily forested, with lower elevation riparian grasslands and higher elevation alpine terrain. Public lands, primarily managed by the U.S. Forest Service, comprise over 95% of the area. The East Fork area exhibits more moderate terrain, with elevations ranging from 1200 to 2800 m. The area is a mix of open grasslands, mid‐elevation rolling hills, and heavily timbered slopes merging into subalpine and alpine areas along the Continental Divide (Figure 1). Lower elevation areas are primarily agricultural land, grassland, shrubland, or forest. Montane grasslands are composed of Idaho fescue (*Festuca idahoensis*), bluebunch wheatgrass (*Pseudoroegneria spicata*), and elk sedge (*Carex geyeri*); shrubland is dominated by sagebrush (*Artemesia tridentata*) or bitterbrush (*Purshia tridentata*), and mixed‐conifer forests are dominated by ponderosa pine (*Pinus ponderosa*) and Douglas‐fir (*Pseudotsuga menziesii*). Higher elevation areas are predominately mesic mixed coniferous forests, which are dominated by lodgepole pine (*Pinus contorta*), grand fir (*Abies grandis*), and subalpine fir (*Abies lasiocarpa*). Public lands are a combination of U.S. Forest Service and state trust lands administered by the Montana Department of Natural Resources and Conservation. Private lands account for roughly 18% of the East Fork and are concentrated in the northwestern portion of the watershed along the Bitterroot River corridor.

Elk are the most abundant ungulate in the study area, with approximately 1000 elk occupying the West Fork area and 3800 elk occupying the East Fork area; elk populations in both areas increased to a peak of approximately 6000 in 2005, then declined due to a combination of female harvest and other factors (Proffitt et al., 2016). Although female harvest was restricted in 2008, the population continued to decline likely due to increases in predation associated with recovering carnivore populations (Eacker et al., 2016). Sympatric ungulate species include mule deer (*Odocoileus hemionus*), white‐tailed deer (*Odocoileus virginianus*), and bighorn sheep (*Ovis canadensis*) in East Fork area, but only small numbers of deer and bighorn sheep occur in the West Fork area. Carnivore species within the study area include black bears (*Ursus americanus*), mountain lions, wolves, and coyotes (*Canis latrans*). Mountain lions occur at a high density of 4.6–5.2/100 km^2^ within the study area (Proffitt et al., 2015) and are the primary predator of calf and adult female elk (Eacker et al., 2017). Wolves recolonized the study area in 2000, and populations steadily increased to a high density of 1.05–1.95/100 km^2^ in 2011 (Bradley et al., 2014).

### 2.2 Prey capture

We captured adult female (AF) elk during the winters of 2011–2013 using a combination of helicopter netgunning and chemical immobilization (Appendix S1). All elk capture and handling followed the requirements of University of Montana Protocol 027‐11MHWB‐042611. A mixture of carfentanil and Xylazine was delivered intramuscularly for sedation (following the Montana Department of Fish, Wildlife and Parks Biomedical Protocol for free‐ranging cervids, a combination of 0.01 mg/kg Carfentanil + 0.1 mg/kg Xylazine). We outfitted elk with Global Positioning System (GPS) collars programmed to collect one location every 30 min or one location every 2 h (model 3300L, Lotek Wireless). Collars were built with a release mechanism programmed to release the collar after 1 year.

### 2.3 Predator capture

We captured four wolves from four different packs during 2008–2014 using a combination of netgunning and darting. Ground captures were conducted with foothold traps designed with offset teeth and rubber‐coated jaws to reduce injury (EZ Grip # 7 double long spring traps, Livestock Protection Company). Aerial captures were conducted by Montana Department of Fish, Wildlife and Parks‐contracted crews using helicopters and dart guns. Wolves were anesthetized using Telazol and handled in accordance with MFWP's biomedical protocol for free‐ranging wolves. Wolves were outfitted with GPS collars programmed to record one location every 2 h (two collars, GPS 700 Iridium, Lotek Wireless) or one location every 3 h (two collars, GPS 7000SW_Argos, Lotek Wireless). We also captured and deployed VHF collars on a total of 34 wolves in 10 wolf packs from 2006 to 2014 and used this relocation information as an external validation of resource selection estimated from GPS‐collar‐derived locations. All wolf capture and handling followed requirements of the Institutional Animal Care and Use Committee for Montana Department of Fish, Wildlife and Parks. We captured 13 mountain lions within the study area in 2016, using trained hounds to tree and chemically immobilize animals using a mixture of ketamine and medetomidine delivered intramuscularly (following the Montana Department of Fish, Wildlife and Parks Biomedical Protocol for free‐ranging felids, a combination of ketamine at 2 mg/kg and medetomidine at 0.75 mg/kg). All mountain lion capture and handling followed the requirements of Montana State University Protocol 2016‐06. We outfitted mountain lions with GPS collars programmed to record one location every 4 h (TGW‐4477‐4, Telonics Telemetry‐Electronics Consultants). Collars were built with a release mechanism programmed to release the collar after 2 years.

### 2.4 Estimating forage quality and risk

**FIGURE 2:**
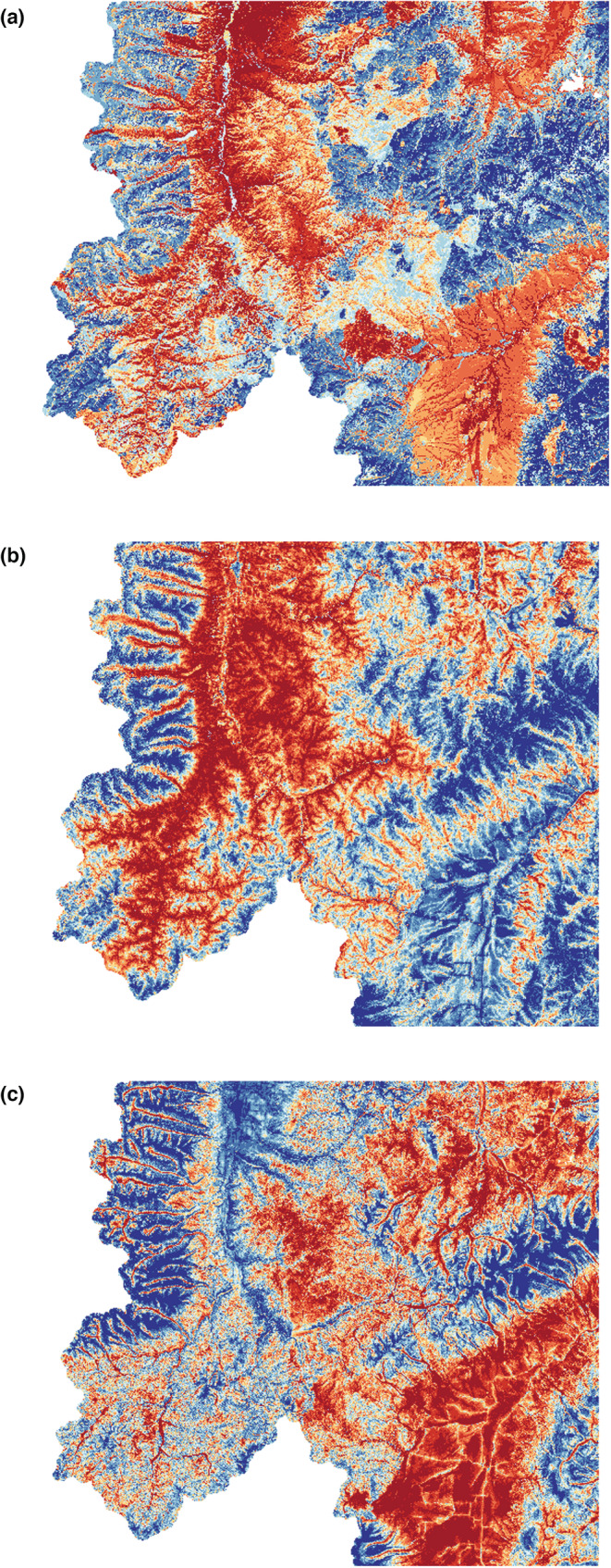
Within the extent of the study area values of summer forage quality (panel a, kcal/g), mountain lion risk (panel b), and wolf risk (panel c) were binned into 10 quantiles with red representing high values and blue representing low values.

We used a previously developed generalized linear model that estimated summer elk forage quality, defined as the kcal of digestible energy (DE) per square meter across the study area (Proffitt et al., 2019), a brief summary of which is included here (Figure 2). This modeling approach first used fecal plant fragment analysis from elk pellet samples to identify important summer forage species; once identified, a spatial model for total DE was developed for these forage species based on sampled vegetation at 752 sites that were stratified across 12 landcover types. At each site, the authors established a 40‐m transect and sampled species composition, percent cover, and dominant phenological stage of each species. To estimate the phenophase‐specific quality of forage species, the sampling protocol collected replicate forage species in each phenological stage and estimated dry matter digestibility using sequential detergent fiber analysis (Wildlife Habitat Nutrition Laboratory). Dry matter digestibility was then converted to digestible energy using methods from Cook et al. (2016). To estimate forage quality within each quadrat at each sampling site, the authors first rescaled percent cover to include the proportion of each forage species in each phenological stage. They then estimated DE of all forage as the weighted mean of the phenophase‐specific DE estimates for each species, weighted by rescaled proportion cover. Last, the authors estimated a mean forage quality (mean kcal/g of forage species) per sampling site based on the estimated forage quality at each of five quadrats along the transect at each sampling site. The final model (based on a stepwise selection process) estimated year‐specific DE as a function of landscape and vegetation characteristics and was then used to predict forage quality across the study area from 2012 to 2015. This estimate of forage quality represented a within‐season average of phenological‐phase‐specific DE, by construction treating it as a fixed metric within each season.

To estimate spatial variation in risk (defined here as the probability of encountering a predator) to elk by wolves and mountain lions, we assumed that wolf and mountain lion risk is proportional to their relative probability of use at the scale of our risk models, the major underlying assumption of this piece of the analysis (Hebblewhite & Merrill, 2007; Kauffman et al., 2007). These, and other, previous studies demonstrate that a significant portion of variation in spatial risk is driven by the resource selection pattern of the large carnivore. For both wolf and mountain lion risk, we developed a summer resource selection function (RSF) model as an index of landscape‐ and local‐scale predation risk for elk (Appendix S1; Figure 2). We included location data collected between June 1 and August 31 (2008–2009 and 2013–2014 for wolves, 2017 for mountain lions). We used a used‐available RSF design to evaluate the strength of selection for landscape covariates by comparing covariate values at wolf or mountain lion GPS locations (used) to random (available) locations sampled from within the population‐level summer ranges (defined as 99% fixed‐kernel isopleths calculated using one location per animal per day) of each predator (Johnson et al., 2006; Manly et al., 2002). In the multiscale terminology of Johnson (1980), we consider this risk model to be a joint second‐ and third‐order RSF because it compares fine‐scale GPS locations to broadscale population‐level summer range availability, such that inferences should reflect concurrent patterns of both second‐order (home ranges within a study area) and third‐order (locations within a home range) selection (Hebblewhite & Merrill, 2007). We validated the mountain lion and wolf RSF models using a fivefold cross‐validation and Spearman rank correlation tests (Boyce et al., 2002) to assess model fit. Notably, our location data for wolves and mountain lions did not temporally overlap elk location data (although they did spatially, Appendix S1); therefore, we used leave‐one‐out cross‐validation of the RSF models for both wolves and lions to determine how well our models predicted the relative probability of selection for individuals not included in the dataset. For wolves, we further conducted external model validation of the wolf RSF by testing the predictive performance of the model using the VHF telemetry data as a testing sample (i.e., the RSF was based on GPS locations alone).

### 2.5 Individual elk responses to forage quality and risk

To assess the presence of trade‐offs between predation risk and forage quality, we evaluated individuals' second order of selection of summer home ranges within population home ranges, and third‐order selection of locations within individual summer home ranges using a used‐available sampling design (Johnson et al., 2006, Manly et al., 2002). We defined summer as July 1–August 31 to exclude any early summer movements associated with movements from calving range to summer range (Cross et al., 2015) and any late summer movements associated with the start of the archery hunting period in early September (Ranglack et al., 2017). We evaluated second‐order selection by comparing individual summer home ranges with the available population‐level (East Fork or West Fork) summer home ranges. We estimated individual and population‐level home ranges using 99% fixed‐kernel isopleths calculated using one location per animal per day using the adehabitatHR package (Calenge, 2006) in the R Programming environment (R Core Team, 2020). We sampled individual home ranges by generating 500 random points within the individual home range polygon. This sample represented the second‐order use locations. We then randomly generated 1000 corresponding available locations within the appropriate population‐level summer home range for each individual to represent the second‐order available set. Previous work on elk has suggested that resource selection may vary throughout the day as animals alternate between different behaviors (e.g., foraging, resting, and hiding; Beyer & Haufler, 1994; Roberts et al., 2017). As our goals were to assess foraging resource selection, for the third‐order analysis, we excluded used locations collected during the warmest time of day (1100–1800 h) when animals are likely to be resting rather than foraging (Appendix S2, which also includes a sensitivity analysis of this censoring; Merrill, 1991). We evaluated third‐order selection by comparing used locations to randomly selected available locations within an individuals' home range at a ratio of 10 available locations per 1 used location.

We evaluated the effects of three covariates representing effects of wolf risk (wolf RSF), mountain lion risk (mountain lion RSF), and forage quality (DE) on summer elk resource selection. Moreover, we included interactions between forage quality and wolf risk and between forage quality and mountain lion risk to evaluate if selection for resources at either the second‐ or third‐order varied in response to predation risk (the trade‐off component required for NCEs). To account for unbalanced sample sizes and different levels of exposure to risk and forage quality within home ranges, we included a random effect (intercept) for each individual elk (Bennington & Thayne, 1994; Gillies et al., 2006; Pendergast & Jane, 1996). Furthermore, to evaluate the evidence for among‐individual heterogeneity in response to variation in forage and risk, to eventually assess the evidence for a relationship between individual trade‐offs and fall body fat and pregnancy rates, and to make better population‐level inferences in the presence of such among‐individual heterogeneity, we incorporated random slopes and interactions for each individual elk (Cooch et al., 2002; Gillies et al., 2006). Our approach also allowed a qualitative assessment of how well population‐level parameters represented a potentially diverse set of individual resource selection patterns.

We estimated the parameters of the resource selection function using the exponential form to link the resource selection function to our covariates, which allowed the models to be estimated using standard logistic regression (Muff et al., 2020). Our model for the relative probability of use for individual *i* at location *j* ($\omega \left(X_{i,j}\right)$) for both second‐ and third‐order selection was as follows:

$$\omega \left(X_{i,j}\right)=\exp\left(\;\gamma_{0}^{i}+\gamma_{\mathrm{DE}}^{i}*{\mathrm{DE}}_{j}+\gamma_{\mathrm{ML}}^{i}*{\mathrm{ML}}_{j}+\gamma_{\mathrm{WF}}^{i}*{\mathrm{WF}}_{j}+\gamma_{\mathrm{DE}*\mathrm{ML}}^{i}*{\mathrm{DE}}_{j}*{\mathrm{ML}}_{j}+\gamma_{\mathrm{DE}*\mathrm{WF}}^{i}*{\mathrm{DE}}_{j}*{\mathrm{WF}}_{j}\right)$$

where DE_j_ = digestible energy at location *j*, ML_j_ = the logarithm of the predicted relative probability of use for location *j* by mountain lions, WF_j_ = the logarithm of the predicted relative probability of use for location *j* by wolves, $\gamma_{0}^{i}$ = the random intercept for individual *i*, and $\gamma_{\mathrm{cov}}^{i}$ = random slopes for individual *i* for covariate cov (DE, ML, or WF) We adopted a Bayesian approach for its utility in modeling random effects, and used hierarchical centering for individual random effects, for example, for random intercepts $\gamma_{0}^{i}$ ~ Normal(β_0_, 100), for random slopes $\gamma_{\mathrm{DE}}^{i}$ ~ Normal(β_DE_, $\sigma_{\mathrm{DE}}$). Population‐level regression parameters (e.g., β_0,_ β_DE_) were assigned vague priors (Normal[0, 2]), as were variances for the distributions of random effects (e.g., $\sigma_{\mathrm{DE}}$ ~ Uniform(0, 2)). We fit a single model for each order of selection and assessed the strength of evidence for each covariate and interaction by evaluating if the credible interval included zero. For population‐level predictions, we built relationships between covariates and relative probability of use with the estimates for the population‐level effects to illustrate the resource selection function. For individual‐level predictions, we built relationships between covariates and relative probability of use using only estimates of individual regression coefficients.

We validated both second‐ and third‐order population‐level RSF models with fivefold cross‐validation following Boyce et al. (2002). We randomly assigned each individual elk to one of the fivefolds, and then iteratively re‐estimated final models with the used and available data of each fold withheld. For each fold of withheld data, we generated predicted values from re‐estimated model coefficients and used percentiles of predicted values in the available sample of withheld data to designate cut‐off values among ordinal bins of habitat suitability, ranked lowest to highest from 1 to 5. We then validated models using a Spearman rank correlation test (*r*_s_) to compare the frequency of withheld used elk locations in each of five bins to each bin's relative ranking (Boyce et al., 2002).

### 2.6 Evaluating the evidence for NCEs: Elk body fat and pregnancy

We sampled adult female elk body condition and pregnancy during the late fall (November 26–December 4) in 2012 and 2013. Different individuals were sampled during each year. We measured chest girth and assessed body condition using a portable ultrasound machine to estimate levels of ingesta‐free body fat following the revised methods of Cook et al. (2010) that included an allometrically scaled MAXFAT index. We assessed lactation status based on the presence of milk in the udder, presence of saliva on the udder, and overall udder size. We determined pregnancy status based on pregnancy specific protein‐B levels in blood serum (Noyes et al., 1996). To evaluate the evidence for NCEs, we then evaluated the association between individuals' trade‐offs for mountain lion risk, wolf risk, and forage quality with their body fat and pregnancy. We used the individual effects estimated from the third‐order resource selection analysis to construct a metric for the magnitude of an individual's trade‐off. We first assumed that the 95% percentile value of digestible energy across the dataset represented high‐quality forage, and then estimated the difference between the relative probability of use at the 95% quantile of predator risk and the relative probability of use at the 5% of predator risk (i.e., positive values indicate the relative probability of use is higher under higher predator risk, negative values indicate the probability of use is higher under low predator risk, or a trade‐off). We then treated these estimated values as explanatory covariates for body fat and the probability of pregnancy, and we predicted that the magnitude of this trade‐off should be negatively associated with body fat and the probability of pregnancy. The individual metrics of trade‐offs used here were based on individual resource selection patterns that were estimated from location information in the summer following physical sampling, that is, after body fat and pregnancy were assessed. The major underlying assumption of this approach is that resource selection patterns of individuals are consistent from year to year, similar to the assumption of a static landscape of risk forced by the temporal disjunction between elk, wolf, and mountain lion location information.

Previous work has established the importance of lactation in determining fall body fat, reflecting the fact that the lactation period for mammals is energetically demanding (Clutton‐Brock et al., 1989; Cook et al., 2013; Parker et al., 2009). Therefore, to account for lactation status and assess the strength of evidence for a relationship with the magnitude of a trade‐off between predator risk and forage quality, we evaluated two models for body fat and the probability of pregnancy that included interactions with lactation status. Given our modest sample size, we used relatively simple models to estimate the relationship between our covariates and the body fat of individual *i* (IFBF_*i*_) and the pregnancy status (PREG_*i*_):

1. Mountain lion risk model:
$${\text{IFBF}}_{i}=\alpha +\beta_{\mathrm{ML}}*{\mathrm{ML}}_{i}+\beta_{\text{LACT}}*{\text{LACT}}_{i}+\beta_{\mathrm{ML}*\text{LACT}}*{\mathrm{ML}}_{i}*{\text{LACT}}_{i}+\epsilon_{i},\epsilon_{i}\sim \text{Normal}\left(0,\sigma^{2}\right)$$
$$\text{logit}\left({\text{PREG}}_{i}\right)=\alpha +\beta_{\mathrm{ML}}*{\mathrm{ML}}_{i}+\beta_{\text{LACT}}*{\text{LACT}}_{i}+\beta_{\mathrm{ML}*\text{LACT}}*{\mathrm{ML}}_{i}*{\text{LACT}}_{i}$$
2. Wolf risk model:
$${\text{IFBF}}_{i}=\alpha +\beta_{\mathrm{WF}}*{\mathrm{WF}}_{i}+\beta_{\text{LACT}}*{\text{LACT}}_{i}+\beta_{\mathrm{WF}*\text{LACT}}*{\mathrm{WF}}_{i}*{\text{LACT}}_{i}+\epsilon_{i},\epsilon_{i}\sim \text{Normal}\left(0,\sigma^{2}\right)$$
$$\text{logit}\left({\text{PREG}}_{i}\right)=\alpha +\beta_{\mathrm{WF}}*{\mathrm{WF}}_{i}+\beta_{\text{LACT}}*{\text{LACT}}_{i}+\beta_{\mathrm{WF}*\text{LACT}}*{\mathrm{WF}}_{i}*{\text{LACT}}_{i}$$

where ${\mathrm{ML}}_{i}$ was the metric of trade‐off for mountain lion risk for individual *i*, ${\mathrm{WF}}_{i}$ was the metric of trade‐off for wolf risk for individual *i*, and ${\text{LACT}}_{i}$ was the lactation status of individual *i*. Intercepts for both body fat and pregnancy models were assigned a Normal(0, 100) prior, and regression coefficients were assigned a Normal(0, 100) prior for the body fat model and Normal(0, 2) for the pregnancy model. We did not include age as a covariate on body fat or pregnancy because previous analysis suggested age and body fat and age and pregnancy were not related in this dataset (Proffitt et al., 2016).

### 2.7 Model estimation

For each component of our analysis, we fit models using the greta package (Golding, 2019) in the R Programming environment (R Core Team, 2020). For resource selection functions at both scales, we treated the variance of the random intercepts as fixed to avoid shrinkage of these effects to the overall mean given the potential for different levels of exposure to habitat conditions, and the likelihoods of the available points were weighted by a factor of 1000 (Muff et al., 2020). Prior to each analysis, all continuous covariates were centered using the mean and standardized using one standard deviation. No pairwise correlation between covariates in our model exceeded 0.50. We summarized inference on estimated parameters by reporting the mean and 90% credible interval from the approximate posterior distribution for the parameter.

## 3 RESULTS

We collected location data from 68 individual female elk, 34 in the East Fork area and 34 in the West Fork area; four individual elk had 2 years of location data. The dataset contained 214,420 individual summer elk locations, with an average of 3335 locations per individual (range = 372–8889 locations). We censored six individuals that did not have accurate time signatures for the GPS locations from the third‐order analysis, resulting in a sample size of 62 individual female elk. The population‐level summer range was 2141 km^2^ and comprised 10% montane riparian areas, 27% grasslands, shrubland, and open woodlands, 34% recently (<16 years) burned forests, and 28% forests with no recent burn history. Using the used‐available datasets, we found that there was a correlation between both mountain lion and wolf risk and digestible energy at both orders of selection (2nd‐order selection: *R* ^2^ = .35 for mountain lions, *R* ^2^ = .15 for wolves; 3rd‐order selection: *R* ^2^ = .34 for mountain lions, *R* ^2^ = .14 for wolves; Appendix S3), which established the potential for trade‐offs between forage quality and risk.

### 3.1 Population‐ and individual‐level responses to forage quality and predation risk

Fivefold cross‐validation of RSF models showed significant correlation between the elk resource selection function model predictions and the locations from withheld elk for both second‐order (*r*_s_ = 1, *p* = .012) and third‐order (*r*_s_ = 1, *p* = .012) selection models.

**FIGURE 3:**
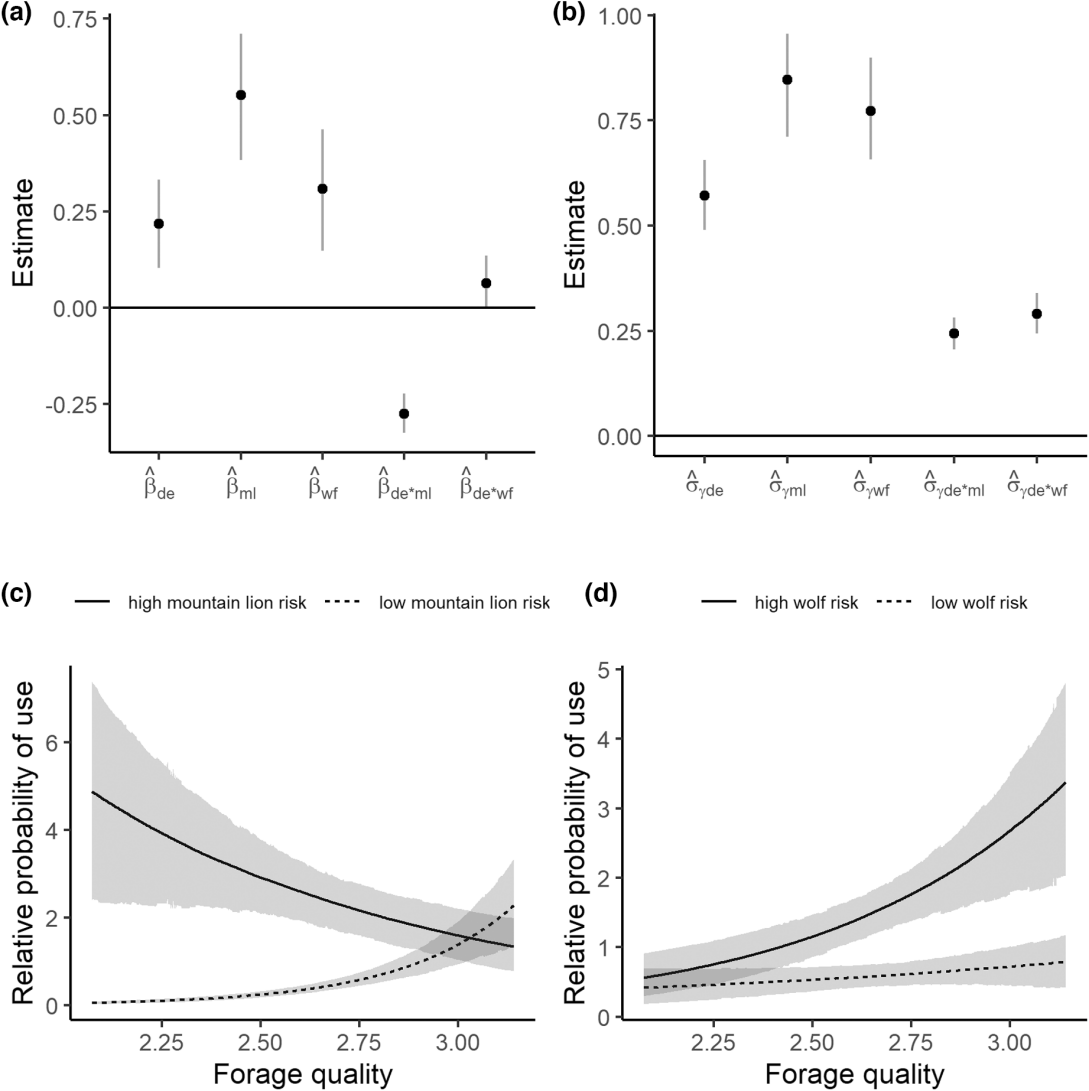
Results of the second‐order elk summer resource selection model and predicted relationships between forage quality, predation risk and resource selection. The top panels illustrate the coefficient estimates (on the log scale) for the fixed effects (panel a) and the estimates of variance components for the random effects (panel b). The dots indicate medians, and the vertical lines indicate the approximate 90% credible interval. The bottom panels illustrate the predicted relative probabilities of selection in response to varying values of digestible energy (DE) for high predator risk and low predator risk for mountain lions (panel c) and wolves (panel d). The dark lines indicate means, and the light gray line indicates an approximate 90% credible interval.

**FIGURE 4:**
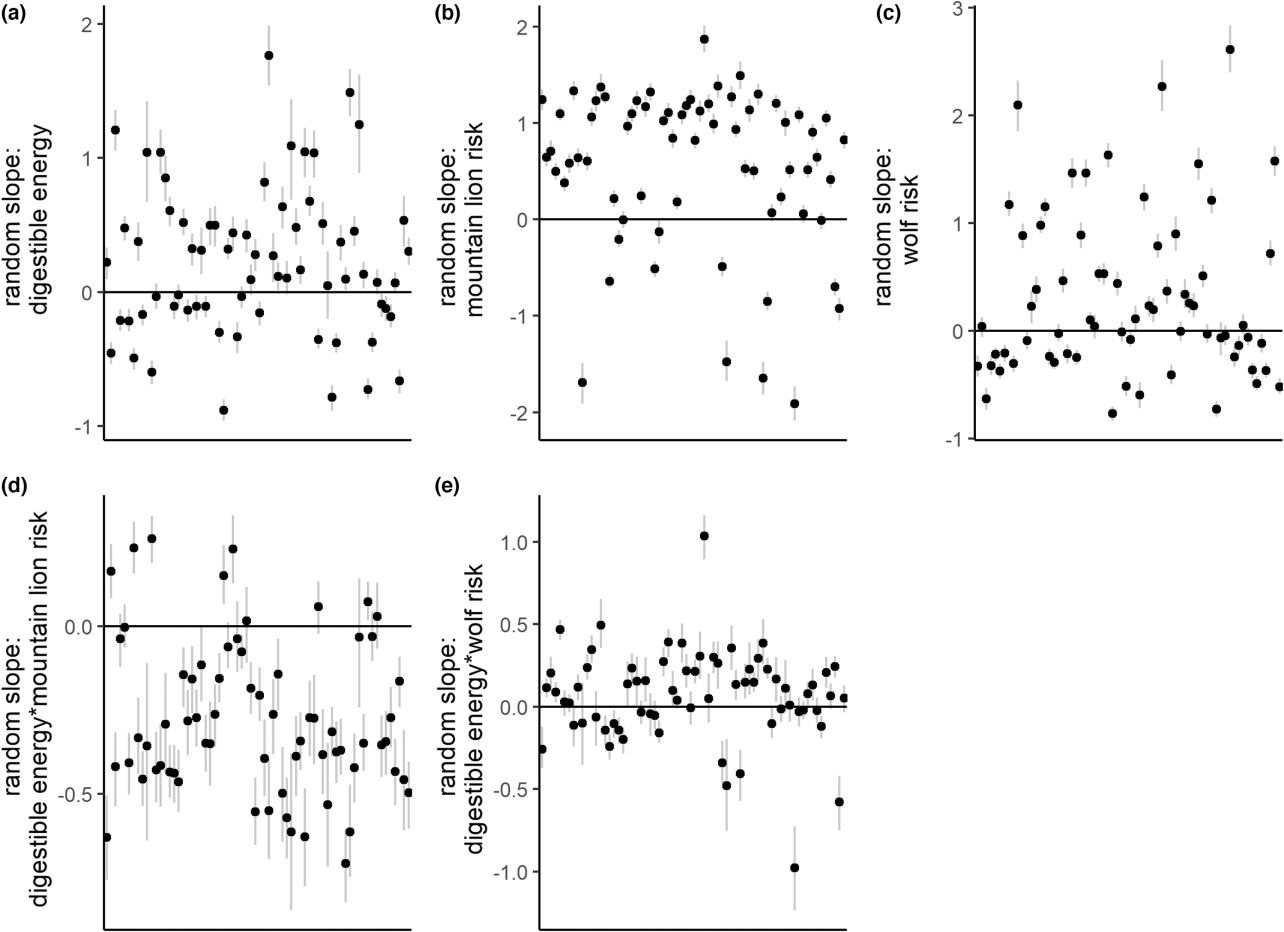
Estimated individual random coefficients for the second‐order resource selection function on the log scale. The *x*‐axes correspond to individual elk, and the *y*‐axes to the estimated regression coefficient. Black dots indicate the mean estimate for the effect, and the light gray lines indicate an approximate 90% credible interval.

**FIGURE 5:**
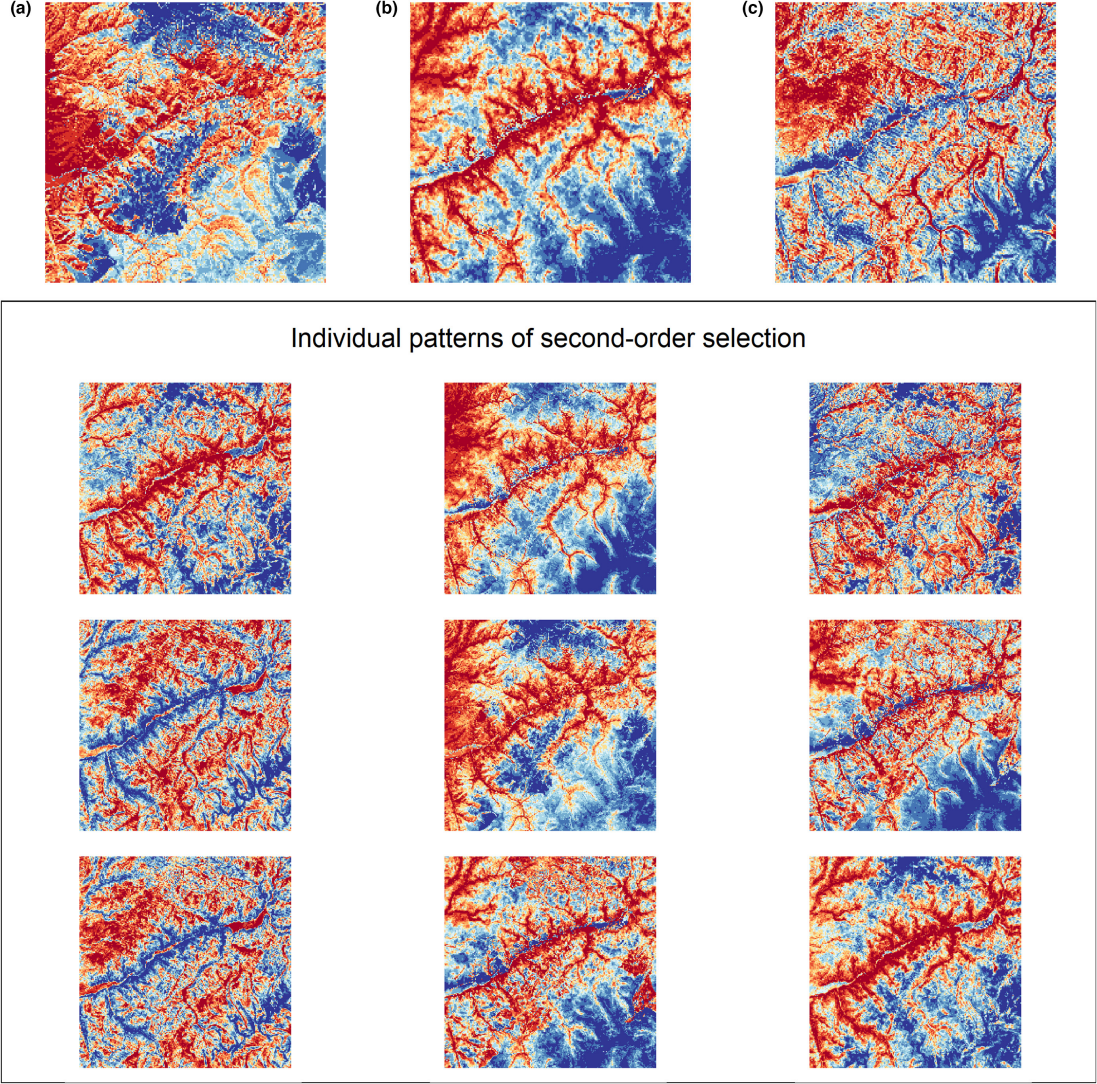
Example of predicted relative probabilities of use for a small sample of individual female elk (*n* = 9). A small section of the study area was used for predictions. Panel a illustrates predicted values of digestible energy, panel b illustrates predicted values of relative probabilities of selection for mountain lions, and panel c illustrates predicted values of relative probabilities of use for wolves. The lower panel illustrates the variety of predicted relative probabilities of selection (2nd order) for elk in response to the underlying landscape of forage quality and risk. To aid illustration, all values were binned into 10 quantiles and coded from low (blue) to high (red).

For second‐order selection for summer home ranges within the population level summer range, we found evidence for a positive population‐level relationship between selection and forage quality ($\hat{\beta }$_de_ = 0.22, 90% credible interval = [0.10, 0.33]) and mountain lion risk ($\hat{\beta }$_ml_ = 0.55 [0.38, 0.71]), and an interaction between forage quality and mountain lion risk ($\hat{\beta }$_de*ml_ = −0.28 [−0.32, −0.22]) such that the relative probability of use of areas with high forage quality decreased under high mountain lion risk, whereas the relative probability of use increased as forage quality increased under low mountain lion risk (Figure 3). In contrast, we found a positive relationship between wolf risk and second‐order selection ($\hat{\beta }$_wf_ = 0.31 [0.15, 0.46]), and evidence for a very weak interaction between forage quality and avoidance of wolf risk ($\hat{\beta }$_de*wf_ = 0.06 [0.00, 0.14]) such that selection for digestible energy increased in association with higher values of wolf risk (Figure 3). We found substantial evidence for among‐individual heterogeneity in resource selection patterns. The variance components for random intercepts and slopes were all estimated to be larger than 0 (Figure 3) and reflected a diverse set of patterns of individual responses to forage quality and risk (Figures 4 and 5).

**FIGURE 6:**
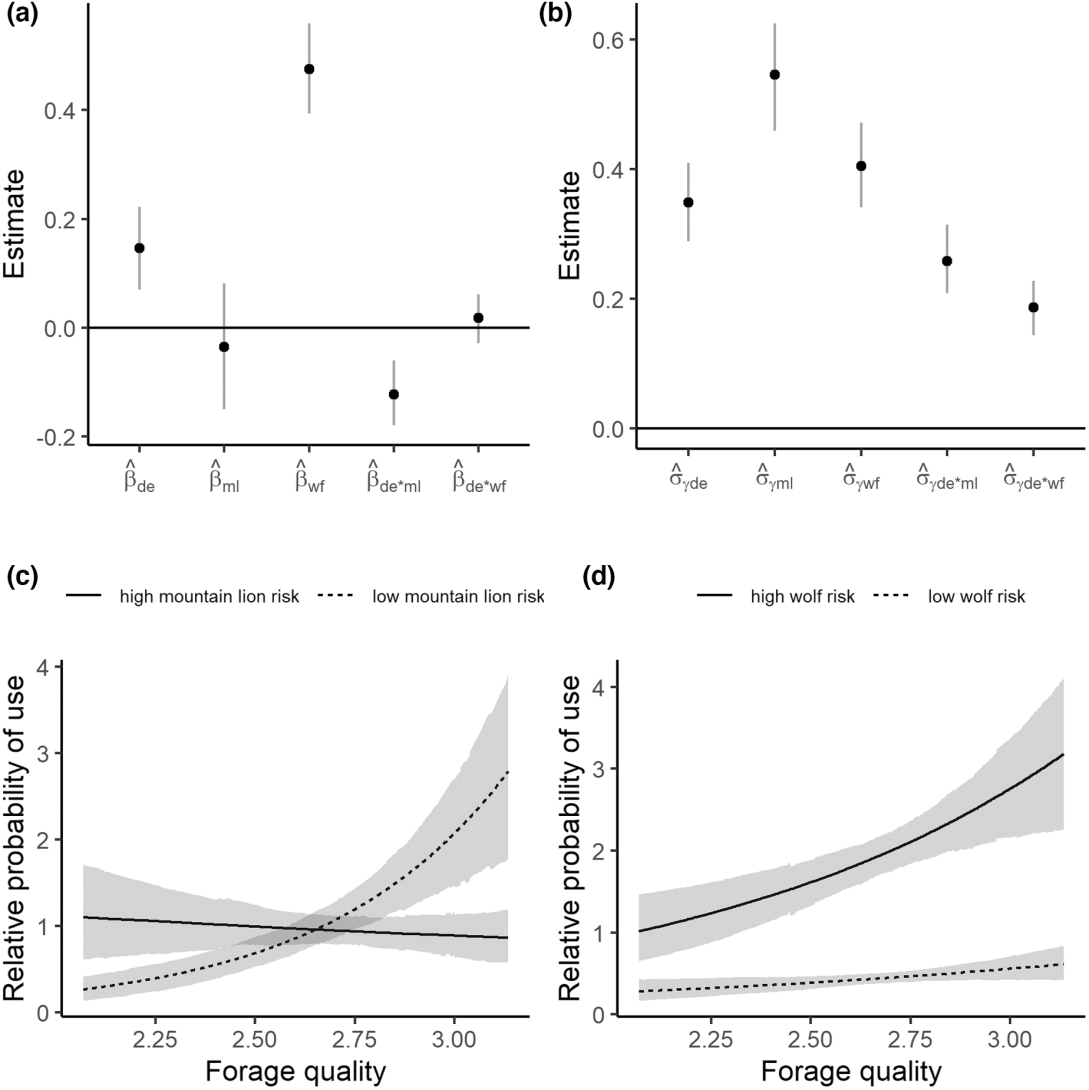
Results of the third‐order elk summer resource selection model, and predicted relationships between forage quality, predation risk and resource selection. The top panels illustrate the coefficient estimates (on the log scale) for the fixed effects (panel a) and the estimates of variance components for the random effects (panel b). The dots indicate medians, and the vertical lines indicate the approximate 90% credible interval. The bottom panels illustrate the predicted relative probabilities of selection in response to varying values of digestible energy (DE) between high predator risk and low predator risk for mountain lions (panel c) and wolves (panel d). The dark lines indicate medians, and the light gray line indicates an approximate 90% credible interval.

**FIGURE 7:**
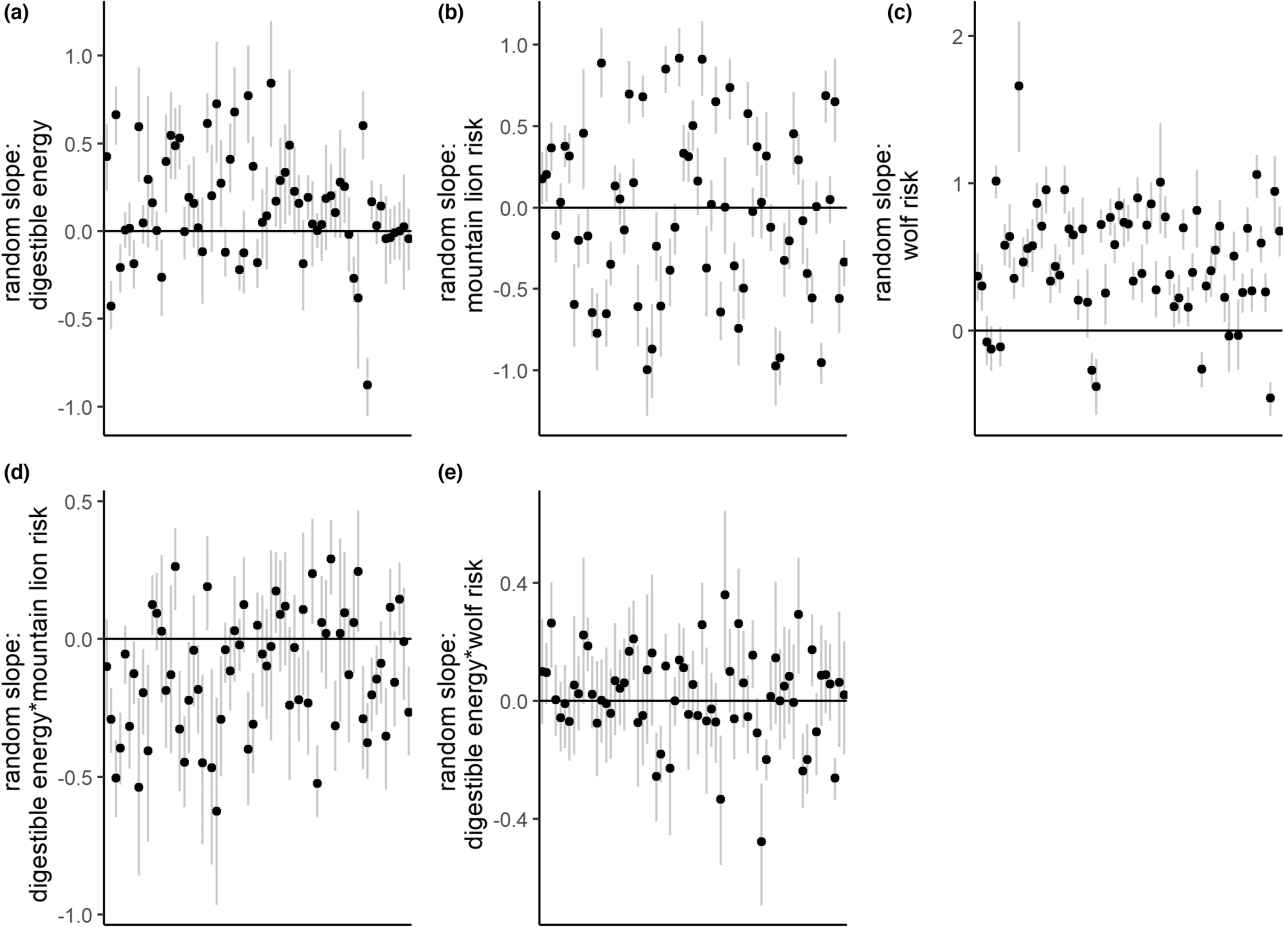
Estimated individual random coefficients (the combination of the fixed effects and random effects) for the third‐order resource selection function on the log scale. The *x*‐axes correspond to individual elk, and the *y*‐axes to the estimated regression coefficient. Black dots indicate the median estimate for the effect, and the light gray lines indicate an approximate 90% credible interval.

**FIGURE 8:**
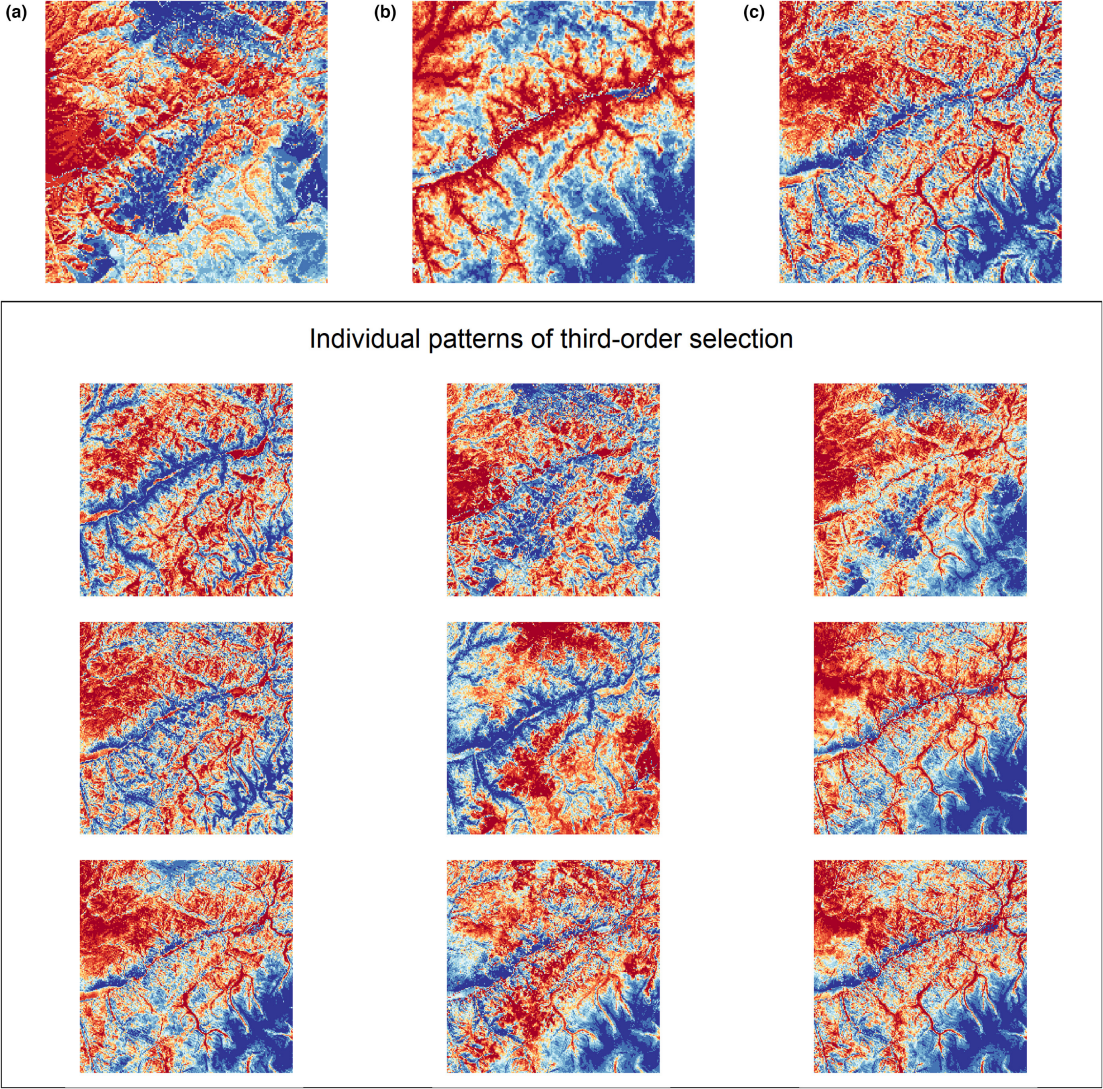
Example of predicted relative probabilities of selection (3rd order) for a small sample of individual female elk (*n* = 9). A small section of the study area was used for predictions. Panel a illustrates predicted values of digestible energy, panel b illustrates predicted values of relative probabilities of selection for mountain lions, and panel c illustrates predicted values of relative probabilities of use for wolves. The lower panel illustrates the variety of predicted relative probabilities of selection (3rd order) for elk in response to the underlying landscape of forage quality and risk. To aid illustration, all values were binned into 10 quantiles and coded from low (blue) to high (red).

At the third‐order of selection for used locations within individuals' home ranges, we found evidence for a pattern between forage quality and mountain lion risk that predicted a decline in selection for higher quality forage in areas with higher mountain lion risk ($\hat{\beta }$_de_ = 0.17 [0.10, 0.25]; $\hat{\beta }$_ml_ = −0.10 [−0.21, 0.02]; $\hat{\beta }$_de*ml_ = −0.10 [−0.16, −0.04]; Figure 6). Similar to second‐order selection, this interaction suggested that the relative probability of use of lower‐quality forage is higher under high mountain lion risk. We found evidence for a relationship between wolf risk and third‐order selection that suggested that selection increased in higher‐risk areas, but no evidence for an interaction between forage quality and wolf risk ($\hat{\beta }$_wf_ = 0.48 [0.40, 0.56]; $\hat{\beta }$_de*wf_ = 0.03 [−0.01, 0.08]; Figure 6) We again found strong evidence for among‐individual heterogeneity in selection (Figure 7) that reflected a diverse set of individual elk responses to forage quality and risk and suggest a range of behaviors from risk‐averse to risk‐tolerant (Figures 7 and 8).

### 3.2 Evaluating the evidence for NCEs: Elk body fat and pregnancy

**FIGURE 9:**
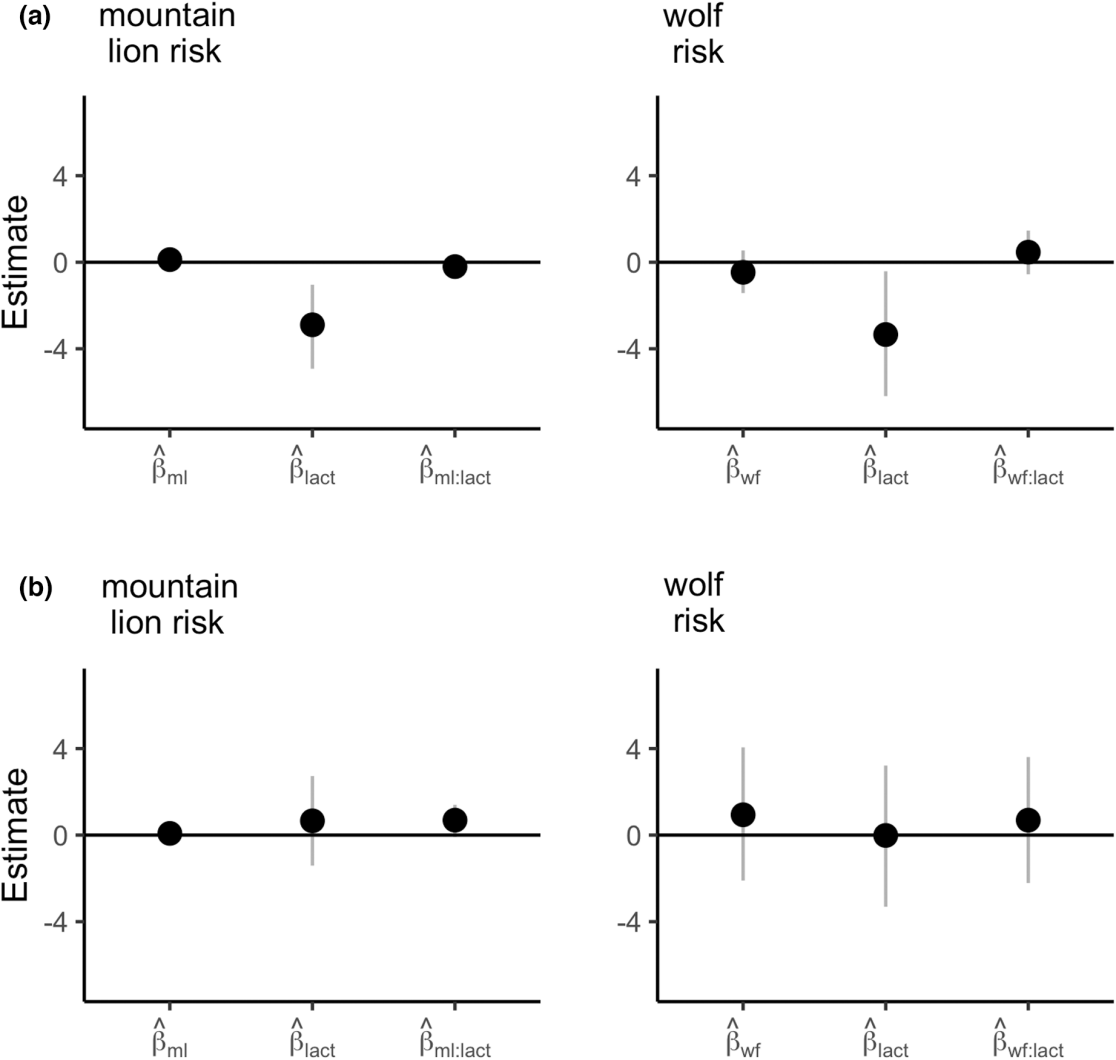
Estimated regression coefficients for models of fall ingesta‐free body fat (IFBF, panel a) and pregnancy (panel b) as functions of individual trade‐offs between selection for forage quality and mountain lion risk (ml) and wolf risk (wf), and lactation status (lact). Individual trade‐offs were estimated as the difference in relative probabilities of use for high‐quality forage between areas of high predator risk and low predator risk. The dots indicate medians, and the vertical lines indicate the approximate 90% credible interval.

A total of 27 animals had fall body fat, pregnancy, and location data collected and were included in the resource selection, body fat and pregnancy analysis. Thirteen animals were sampled in 2012, and 14 animals were sampled in 2013 (Appendix S1). Thirteen of 27 animals were lactating. Median body fat was 7.5% and the pregnancy rate was 76%. We found strong evidence that lactation status was negatively related to fall body fat (ML model: ${\hat{\beta }}_{\text{lact}}$ = −2.89 [−4.92, −1.04]; WF model: ${\hat{\beta }}_{\text{lact}}$ = −3.34 [−6.19, −0.42]), but not the probability of pregnancy (ML model: ${\hat{\beta }}_{\text{lact}}$ = 0.66 [−1.41, 2.73]; WF model: ${\hat{\beta }}_{\text{lact}}$ = −0.02 [−3.31, 3.22]; Figure 8). After accounting for lactation status, we found no strong evidence that the magnitude of individual trade‐offs between forage quality and predation risk were related to either body fat (ML model: ${\hat{\beta }}_{\mathrm{ml}}$ = 0.12 [−0.08, 0.31], ${\hat{\beta }}_{\mathrm{ml}:\text{lact}}$ = −0.20 [−0.76, 0.37]; WF model: ${\hat{\beta }}_{\mathrm{wf}}$ = −0.47 [−1.42, 0.54], ${\hat{\beta }}_{\mathrm{wf}:\text{lact}}$ = 0.47 [−1.42, 0.54]) or the probability of pregnancy (ML model: ${\hat{\beta }}_{\mathrm{ml}}$ = 0.08 [−0.11, 0.27], ${\hat{\beta }}_{\mathrm{ml}:\text{lact}}$ = 0.69 [−0.08, 1.40]; WF model: ${\hat{\beta }}_{\mathrm{wf}}$ = 0.94 [−2.10, 4.06], ${\hat{\beta }}_{\mathrm{wf}:\text{lact}}$ = 0.69 [−2.21, 3.61]; Figure 9).

## 4 DISCUSSION

Behavioral adaptations of prey to predation risk reflect interactions between landscape structure, scale, and style of predation (Atwood et al., 2009; Creel et al., 2005; Kittle et al., 2008; Miller et al., 2014). We found strong evidence for population‐level trade‐offs between mountain lion risk and selection for home ranges and for locations within home ranges with high‐quality forage, and no evidence for population‐level trade‐offs between wolf risk and selection for high‐quality forage. These population‐level patterns were coupled to substantial among‐individual heterogeneity in resource selection. We found that the diversity of individual resource use patterns included risk‐averse strategies with anticipated trade‐offs to forage quality, as well as risk‐tolerant strategies that suggested resource use increased in areas with high predator risk. We found no evidence in our west‐central Montana study area that the magnitude of individual elk trade‐offs between predation risk and forage quality translated into variation in two key physiological parameters related to reproduction.

The predator‐sensitive foraging response to predation risk can take the form of temporal avoidance (risky times) or spatial avoidance (risky places), and empirical evidence suggests that in multi‐predator systems, the form of behavioral response depends on the style of predation (Miller et al., 2014). Prey can spatially respond to ambush‐style predators by avoiding risky places; however, they cannot respond spatially to cursorial predators and, instead, adapt by avoiding areas where predators are present during risky times. The multidimensional habitat domain (the movement patterns of predator and prey throughout the environment) implied by this diversity of antipredation responses suggests that prey may be limited in access to resources due to one type of predation, but unable to spatially adapt to other types and, therefore, not be limited in spatial access to resources. Assuming that the resource use patterns of prey reflect a stationary (static) landscape of risk is misguided. Decades of work on the behavioral responses of predators and prey to one another suggests that a predator response to shifting distribution of prey is largely dependent on the space use patterns of both predator and prey and is best considered as a dynamic (not stationary) behavioral response (Laundré, 2010; Sih, 1984). The assumption that prey can spatially respond to predators through avoidance is conditioned on a narrow habitat domain for the predator, not conditioned on the assumption of a stationary landscape of risk (Schmitz, 2017; Schmitz et al., 2004; Smith et al., 2019). The population‐level results from our study are consistent with this conceptualization, as elk avoided areas of high risk from an ambush predator with a narrow habitat domain (mountain lions) but did not avoid areas selected by a coursing predator (wolves) due to the inability to spatially respond to the more mobile predator. Our results suggested that elk selected for areas of highest wolf risk, which may actually have been an artifact of wolves selecting for areas of highest elk use, that is, the association we estimated was driven by a wolf response to elk, rather than an elk response to wolves, a distinction that requires more work to understand. We also note that the sample size of individual wolves upon which the resource selection was modest (*n* = 4), and, although the resource selection validated very well against other wolves in other packs in other years, these patterns could be the result of an incomplete understanding of wolf resource selection patterns.

Care must be taken when interpreting the fixed effects from a mixed‐effects model as representing the population‐level response (Muff et al., 2016). Although the fixed effects do not represent a true marginal model, they are an approximation of the among‐individual average response to the underlying landscapes of forage and risk. The extent to which predictions from fixed effects are accurate and reliable depends strongly on the degree to which this average response reflects the diversity of underlying individual‐specific responses (Russell et al., 2015). We suggest that the consequences of over‐interpreting population‐level effects for resource selection are relatively mild; after all, those effects represent an average of resource use patterns among individuals, and a simultaneous examination of fixed and random effects (as done here) provides enough context to weigh how well the population‐level average reflects individuals. The consequences of then interpreting these population‐level responses in terms of fitness‐related trade‐offs and evidence for NCEs are likely more severe, given that among‐individual heterogeneity in life‐history characteristics should generate a variety of different balances between access to high‐quality forage and predation risk, that is, trade‐offs inherently exist at the individual level (Gaynor et al., 2019). For example, although the average population‐level responses to risk documented here are consistent with a stalking/cursorial predator paradigm, enough among‐individual heterogeneity exists to suggest that paradigm does not apply to all individuals, a disjunction that we speculate reflects latent differences among individuals.

Fundamental to the detection of trade‐offs in resource selection is the idea that selection coefficients should reflect the underlying availability of resources or exposure to predation risk (e.g., the strength of selection against areas with mountain lion risk are stronger in areas with higher overall mountain lion risk; Mysterud & Ims, 1998). Consequently, trade‐offs may only be detectable at high levels of availability or exposure. We speculate that part of this pattern is also due to the lack of variation in exposure to risk among individuals (i.e., most individuals had very comparable ranges of exposure to mountain lion and wolf risk) and the overall high risk of predation in this system from restored populations of predators (Eacker et al., 2016). Regardless, such among‐individual differences in behavioral responses have significant consequences for the assessment of the NCE hypothesis. Small sample sizes, or analyses that do not explicitly account for among‐individual differences, have a high potential for spurious correlations in large mammal studies if estimated population‐level responses do adequately capture the variation in the responses of individuals, the level at which trade‐offs occur. This problem is further complicated by additional unmodeled sources of heterogeneity in resource selection patterns. Prior work has demonstrated that heterogeneity in movement patterns arises from differences among populations, sex, and ecological dynamics (e.g., variation induced by density‐dependent process or predation; Anderson et al., 2005; McCorquodale, 2003; McLoughlin et al., 2010; Peterson & Weckerly, 2017).

Even if an analysis is at the level of an individual trade‐off, and evidence suggests an individual prey avoids high‐quality resources if those resources are associated with higher predation risk, it does not immediately follow that such avoidance results in a nutritional limitation. Prior work has convincingly demonstrated that access to high‐quality forage has important consequences for body condition and demographic rates for ungulates (Cook et al., 1996, 2004; Merrill & Boyce, 1991; Parker et al., 2009). Due to mechanical limitations (“gut‐fill”, or restriction of intake due to rumen volume) or chemical limitation (e.g., low nitrogen levels in forage resulting in diminished digestion of cellulose), ruminates cannot make up for poor‐quality forage by simply increasing consumption (Owen‐Smith, 1988; Roy et al., 1977). However, a lack of access to the highest‐quality resources does not necessarily result in a body condition that cannot meet the demands of reproduction, over‐winter survival, or offspring provisioning in the spring. There is a minimum amount of digestible energy required to meet the demands on elk in the late summer, and approximately equal body condition can be achieved by a range of forage qualities, although it may take longer to achieve at lower forage quality (Cook et al., 2004). Thus, the spatial avoidance of a particular type of predator and associated trade‐off with the highest forage quality does not necessarily translate into diminished body condition. Our finding that individual‐level elk selection reflecting trade‐offs between mountain lion risk and forage quality did not translate into a relationship with body fat or probability of pregnancy may reflect this concept, although we acknowledge that our power to detect such effects was limited due to small sample size.

The predator‐sensitive foraging mechanism underlying the NCE hypothesis rests on a chain of logic to connect patterns of space use to population demography. Although we found evidence for a potential trade‐off between forage quality and risk from an ambush predator, we failed to find evidence that the trade‐off manifested in two key metrics related to population dynamics. We acknowledge that the sample sizes for the pregnancy and body fat analyses, coupled to the use of predicted metrics across several levels of our analysis, likely rendered this a low‐power assessment of the NCE hypothesis. However, our work highlighted how this conclusion is the aggregated result of a complex interplay of extrinsic and intrinsic spatial relationships between forage and risk and illustrates the challenges of assessing meaningful biological consequences of potential trade‐offs. From a management perspective, the presence or absence of NCEs matter for elk population management, specifically when elk populations are under objective or there is concern over a small population of elk. In this scenario, the most productive management action to take would depend on the extent to which elk nutritional condition and pregnancy rates are influenced by habitat productivity or nutritional carrying capacity, an understanding of what factors were associated with a risk‐averse foraging strategy, and finally the extent to which this variation in vital rates actually affects population dynamics (Sheriff et al., 2020). If habitat and poor forage quality are limiting elk population productivity, habitat treatments to increase elk forage quality (such as, perhaps, large‐scale fire mosaics [Proffitt et al., 2019]) may be a productive approach to increasing elk productivity. However, this will only be successful to the extent that NCEs are not going to override the effect of forage improvements by limiting elk access to forage for enough of the population to have an impact. If NCEs are affecting elk access to forage and thereby limiting population productivity, reducing the density of large carnivores should result in elk population growth, if it is possible for a long enough period over a big enough scale. In our study area, there is concern over the declining elk population in part of this area (Eacker et al., 2017; Proffitt et al., 2016). Because NCEs do not appear to be a major factor, habitat treatments to improve elk forage on elk summer ranges could be a productive management goal for attempting to increase elk productivity, in addition to managing for lower carnivore densities to reduce the direct effects of predation.

There are several limitations of our study that have implications both for the interpretation of our results and future work evaluating behavioral responses to predation risk. First, we used predicted relative probabilities of use for mountain lions (from collar data that did not overlap the time period of the collar data from elk) and wolves as metrics of spatial risk. This rests on four fundamental, linked assumptions that require future work to address: (1) that space use patterns of predators are a proxy for the risk of predation (DeCesare, 2012; Hebblewhite et al., 2005), (2) that these space use patterns translate into equal risk of predation at all times of the day when elk are foraging (Kohl et al., 2019), (3) predator space use and risk of predation do not vary across years. Second, our data did not allow us to address the short‐term behavioral response of elk to the presence of wolves in the immediate area. This could result in changes to foraging patterns such that intake rates are diminished and result in nutritional limitation with potential population‐level consequences (Christianson & Creel, 2010; Winnie & Creel, 2017), particularly given the importance that variation in movement patterns among individuals may have in determining population‐level responses (Jolles et al., 2020). Third, our metrics for forage quality and risk are the modeled result of resource selection functions that incorporated physiographic and habitat covariates (e.g., elevation, slope, normalized difference vegetation index [NDVI]) that help explain elk resource selection in the absence of risk from predation. This has the potential to generate spurious associations between modeled covariates in studies that use risk metrics based on such habitat characteristics (Moll et al., 2017; Prugh et al., 2019), and our results should be placed in the larger context of future work understanding the spatial response of prey to predators. Finally, we note that we included predicted values as explanatory variables at two levels of our analysis: using predicted relative probabilities of use of predators as explanatory variables for second‐ and third‐order selection by elk and predicted metrics of trade‐offs as explanatory variables for variation in body fat and the probabilities of pregnancy. The lack of integration of prediction error into our analysis likely results in over‐confidence in coefficient estimates. In addition to providing necessary context to interpret our results, these limitations also reflect the practical challenges of trying to assess behavioral responses to predation and their implications for population dynamics over broad temporal and spatial scales. As we move toward a coherent framework in which to understand the diversity of ways that predators can impact prey populations, these difficulties point to the need for large‐scale, integrated studies that simultaneously monitor the full scope of the interaction between predator and prey population dynamics.

## AUTHOR CONTRIBUTIONS

**John Terrill Paterson:** Data curation (equal); formal analysis (lead); methodology (lead); writing – original draft (lead); writing – review and editing (lead). **Kelly M. Proffitt:** Conceptualization (lead); formal analysis (supporting); project administration (lead); writing – original draft (supporting); writing – review and editing (supporting). **Justin A. Gude:** Conceptualization (lead); writing – original draft (supporting); writing – review and editing (supporting). **Nicholas J. DeCesare:** Conceptualization (lead); writing – original draft (supporting); writing – review and editing (supporting). **Mark Hebblewhite:** Conceptualization (lead); writing – original draft (supporting); writing – review and editing (supporting).

## FUNDING INFORMATION

This project was funded by the sale of Montana hunting and fishing licenses and funds from Federal Aid in Wildlife Restoration Grant W‐163‐R‐1 to Montana Fish, Wildlife and Parks. Funding was also provided by Rocky Mountain Elk Foundation, Safari Club International Foundation, the Shikar‐Safari Club International Foundation, the Pope and Young Club, McIntire‐Stennis Foundation (USDA) and private donations from individuals in the community.

## CONFLICT OF INTEREST

The authors declare no conflicts of interest.

## PERMITS

Animal handling was approved through the University of Montana IACUC 027‐11MHWB‐042611 and Montana State University IACUC Protocol 2016‐06.

## Supporting information


Appendix S1–S3

## Data Availability

Second‐order and third order elk RSF data sets: https://doi.org/10.5281/zenodo.6896299.
