# Supplementary material for: Evaluating the summer landscapes of predation risk and forage quality for elk (Cervus canadensis)
Source: Ecol Evol. 2022 Aug 11;12(8):e9201. doi: 10.1002/ece3.9201 (PMC9366754; doi:10.1002/ece3.9201)
Supplement: Supplementary file 1 — Appendix S1–S3 [file ECE3-12-e9201-s001.docx]

Appendix S1. Elk sample information, and mountain lion and wolf resource selection function development.

*Elk sample information*

We estimated models for summer second and third-order resource selection for adult female elk, and a model assessing the strength of evidence for a relationship between body fat, pregnancy and adult female elk resource selection (NCEs). Due to sampling problems and inference the sample sizes varied between these three models (Table A1).

|  | | Table A1. Sample information for second-order selection, third-order selection, and pregnancy. | | | | | |
| --- | --- | --- | --- | --- | --- | --- | --- |
| **Model** | **Years** | | **Number of animals** | **Range of location dates or sampling dates** | **Minimum number of locations per animal** | **Maximum number of locations per animal** | **Median number of locations per animal** |
| Resource selection  functions | 2012, 2013 | | 68 (second-order), 64 (third-order) | July 1 to August 31 | 372 | 3335 | 8889 |
|  |  | |  |  |  |  |  |
| NCEs | 2012, 2013 | | 27 | November 6 – December 4 | NA | NA | NA |

*Mountain lion and wolf resource selection function development.*

A key component of our analysis was the use of predicted relative probabilities of selection from mountain lions and wolves as covariates to explain patterns of 2^nd^ and 3^rd^ order selection for elk, based on resource selection modeling under the used-available design. In the most general terms, space use of these three species overlapped in our study area (Figure A1).

Figure A1. GPS locations in the study area for elk (panel a), wolves (panel b), and mountain lions (panel c).


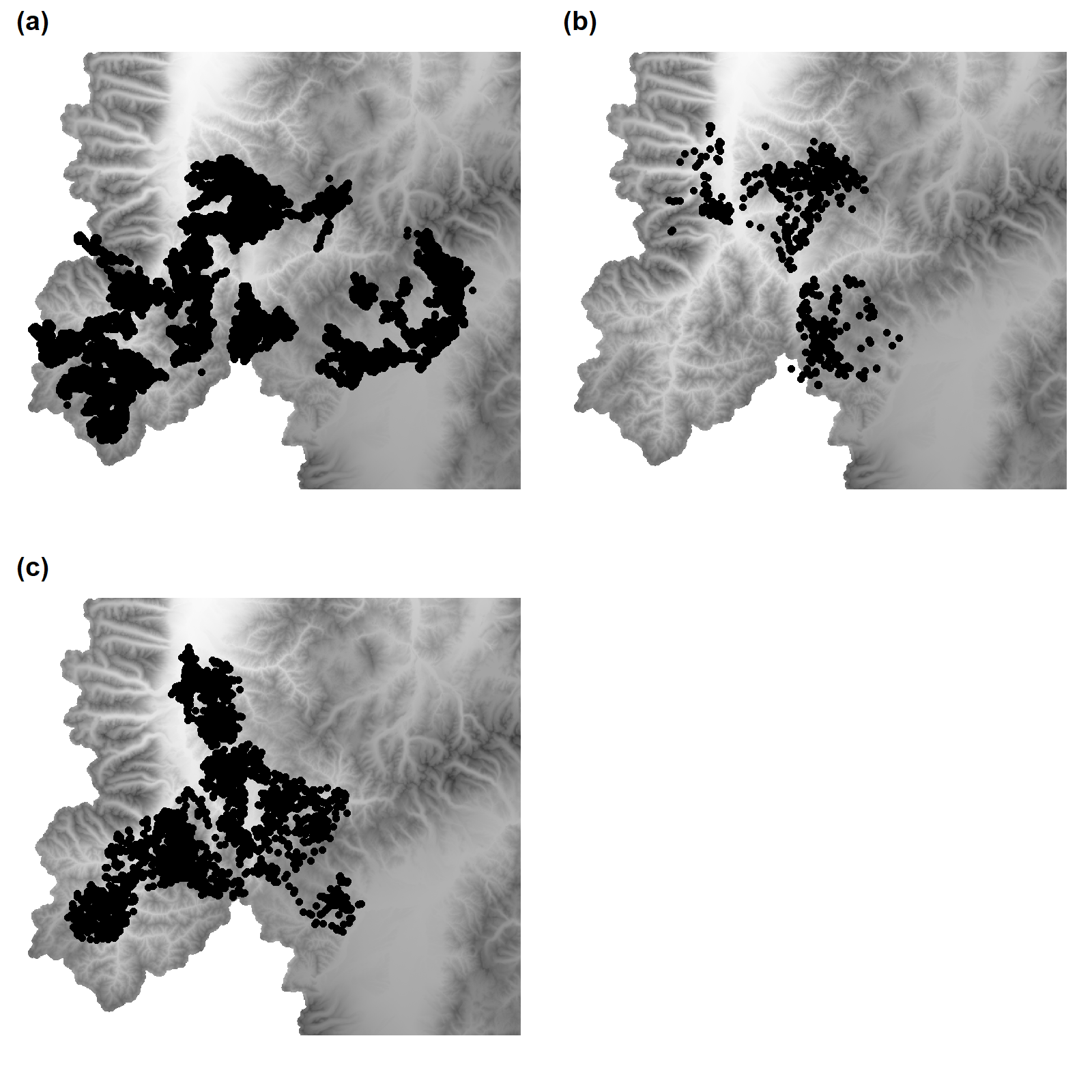


However, the temporal windows of these GPS locations for the three species did not completely align for elk and wolves (elk summer locations from 2012 – 2013; wolf summer locations from 2008, 2009, 2013, and 2014), and did not align at all for elk and mountain lions (mountain lion summer locations from 2017) (Table A2.)

| Table A2. GPS location summaries for the three species. | | |
| --- | --- | --- |
| **Species** | **Year** | **Total points in the location record** |
| elk | 2012 | 95918 |
|  | 2013 | 130842 |
| wolves | 2008 | 65 |
|  | 2009 | 504 |
|  | 2013 | 1140 |
|  | 2014 | 37 |
| mountain lions | 2017 | 7524 |

Interannual variation in resource selection patterns of top predators like wolves and mountain lions is seldom acknowledged, let alone quantitatively assessed (Uboni et al. 2015). For wolves, there is some work suggesting that patterns of selection may change over time or for individuals as a response to the complicated interplay of changing landscape characteristics and prey population dynamics (Uboni et al. 2015). The current state of understanding for mountain lions similarly suggests some variation in resource selection among years and individuals, although general patterns of resource selection for mountain lions are consistent enough to predict patterns of selection across individuals and years (Robinson et al. 2015, Benson et al. 2016, Johnson et al. 2017). Therefore, in addition to developing resource selection functions for wolves and mountain lions, we also wanted to assess how well the estimated model of resource selection could predict patterns for individual wolves and mountain lions (i.e., how general the model was) to assess if the estimated patterns could reliably predict patterns across space and time (wolves), or space alone (mountain lions). For mountain lions, we were not able to assess interannual variation in resource selection patterns, and the generalizability of selection patterns across years remains an assumption of our analysis.

**Methods**

Wolf collaring

We deployed 4 GPS collars on wolves within the study area from August 2008 – 2014; two GPS 7000SW-Argos and two GPS 7000 iridium (LOTEK, Aurora, ON, Canada). GPS iridium collars were programmed to take 1 location every 2 hours, and uploaded data daily to a LOTEK server. GPS 7000-Argos GPS collars were programmed to take 1 location every 3 hours and GPS location data were transmitted to Argos satellites during a 6-hour period once every 10 to 14 days. Additionally, we captured and deployed VHF radiocollars on a total of 34 wolves in 10 wolf packs within and adjacent to the study area from 2006 – 2014. Using VHF radiocollars, we radiocollared an average of 3.7 wolves/pack. Location of VHF collars were monitored 1-3 times per monthly from fixed-wing aircraft. All wolf capture and handling followed requirements of the Institutional Animal Care and Use Committee for Montana Department of Fish, Wildlife and Parks.

Mountain lion collaring

We deployed 13 GPS collars on mountain lions captured within the study area in 2016 (TGW-4477-4, Telonics Telemetry-Electronics Consultants, Mesa, Arizona, USA). GPS collars were programmed to record 1 location every 4 hours and uploaded data daily to the Telonics server. Collars were built with a release mechanism programmed to release the collar after 2-years. All mountain lion capture and handling followed requirements of Montana State University Protocol 2016-06.

Resource selection modeling

We estimated resource selection using the standard used-available resource selection design (Manly et al. 2007). We modeled the relative probability of use at any point *j* for individual *i* ($\omega\left( X_{i,j} \right)$) using the exponential link to estimate the effect of physiographic and environmental covariates on the relative probability of use in a mixed-model framework, i.e.,

$\omega\left( X_{i,j} \right)=exp(\boldsymbol{\beta}^{\boldsymbol{T}}\boldsymbol{x}_{\boldsymbol{i,j}}\boldsymbol{+}\boldsymbol{\gamma}^{\boldsymbol{T}}\boldsymbol{u}_{\boldsymbol{i,j}})$.

where $\boldsymbol{x}_{\boldsymbol{i,j}}$ is the vector of covariates for fixed effects, $\boldsymbol{u}_{\boldsymbol{i,j}}$ is the vector of covariates for random effects, $\boldsymbol{\beta}^{\boldsymbol{T}}$ is the transposed vector of regression coefficients for fixed effects, and $\boldsymbol{\gamma}^{\boldsymbol{T}}$ is the transposed vector of regression coefficients for the random coefficients. This approach allows the use of conventional logistic regression software to estimate the regression coefficients, although we note that the results should not be interpreted as if it were an actual logistic regression (Johnson et al. 2006, Manly et al. 2007). For mountain lions and wolves, we included a random intercept to help account for unbalanced sample sizes among individuals (Fieberg et al. 2010).

To construct the used-available data set for summer resource selection functions for wolves and mountain lions, we first filtered all locations to only include those from June, July and August. Next, we calculated separate population-level summer ranges for each species by randomly sampling the location information to one location per individual per day, and then estimating a 99% kernel home range using the adehabitat package in R (Calenge 2009). Finally, we generated the available points by selecting 10 random locations per used location from within the population-level summer range; this value was chosen after initial model estimates suggested that coefficients estimates stabilized at this ratio. In the multi-scale terminology of Johnson (Johnson 1980), we consider this risk model to be a joint second- and third-order RSF because it compares fine-scale GPS locations to broad-scale population-level summer range availability, such that inferences should reflect concurrent patterns of both second-order (home ranges within a study area) and third-order (locations within a home range) selection (Hebblewhite and Merrill 2007).

We used a Bayesian approach to facilitate the use of random effects and to be consistent in interpretations with our main analysis. Models were estimated using the greta package (Golding 2019) in the R programming environment (R Core Team 2018).

Covariates

We developed a suite of spatial covariates based on previous wolf resource selection studies in the Northern Rockies (Hebblewhite et al. 2005, Bergman et al. 2006, Kauffman et al. 2007, Hebblewhite and Merrill 2008). Covariates reflected variation in topographic features, human activity, landcover, and vegetation productivity (see Table A3 for details). Topographic covariates evaluated included elevation, slope and slope variance. Specifically for mountain lions, we included a quadratic term for slope as this has been previously shown to be important for mountain lion resource selection elsewhere in Montana (Blake et al. 2014). We further developed two indices of human activity: distance to nearest motorized road and distance to development (defined here as the aggregated result of all levels of development under the NLCD classification). Because previous studies showed distance to forest edges and water was important to explain wolf resource selection (Bergman et al. 2006, Hebblewhite and Merrill 2008), we developed a distance to forest cover and distance to water covariate. For all 4 distance-to variables, we considered that the effects of these covariates likely attenuated in a non-linear fashion and, therefore, used a log transformation of the covariate prior to modeling. We developed two additional metrics of landcover and vegetation productivity: vegetation cover type and average greenness as indexed by NDVI. Vegetation cover type was categorized as either forested or not (the aggregate of all other classes) based on the 2011 National Landcover Database landcover model. Finally, we assumed that values of the normalized difference vegetation index (NDVI) derived from the moderate resolution imaging spectroradiometer (MODIS) Terra satellite represented primary production on the landscape, and served as a proxy for annual forage productivity (Pettorelli et al. 2011). We used the 8-day surface reflectance images with 250m resolution (MODIS product MOD09Q1) to calculate NDVI values on a per-pixel basis across the study area and through time (courtesy of the NASA Land Processes Distributed Active Archive Center (LP DAAC), USGS/Earth Resources Observation and Science (EROS) Center, Sioux Falls, South Dakota, <https://lpdaac.usgs.gov/data_access/data_pool>). We then calculated the time-integrated value of NDVI by summing the differences of each value and the value at the start of the growing season to account for the effects of canopy cover. All covariates were centered (using the mean) and standardized (using one standard deviation) prior to estimation.

| Table A3. Spatial GIS covariates developed for spatial modeling in the Southern Bitterroot Elk study area. | | |
| --- | --- | --- |
| **Covariate Name** | **Description/Source** | **Reference** |
| Elevation (m) | Digital elevation model obtained from USGS | DEM courtesy of the U.S. Geological Survey |
| Slope (degrees) | Slope derived from the USGS DEM model | DEM courtesy of the U.S. Geological Survey |
| Slope variance | Calculated at the 100m scale from the 30m USGS dem | DEM courtesy of the U.S. Geological Survey |
| Landcover type | Categorized as 1 = Forest, 0 = Other | 2011 National Landcover Database |
| Distance to forest cover (km) | Forests were defined in Landcover above, and then Euclidean distance in km from forest edges were calculated with 0km the value if inside a forest. | (Bergman et al. 2006, Hebblewhite and Merrill 2008) |
| Distance to motorized roads (km) | Motorized roads were defined as roads open to any type of motorized use during any portion of the year. | Road data was taken from Montana Department of Transportation, the USFS, the U. S. Census Bureau TIGER product, and the Montana Spatial Data Infrastructure. |
| Distance to water (km) |  | Hydrology layers were taken from Idaho Department of Water Resources and the Montana Spatial Data Infrastructure.. We included all streams, rivers and lakes as water sources. |
| Distance to development (km) | Landcover was defined through the 2011 National Landcover Database, with developed areas defined here as the aggregated result of all level of development. A Euclidean distance was calculated in km. | 2011 National Landcover Database |
| NDVI (Normalized Difference Vegetation Index) | MOD09Q1 – 250m resolution; average NDVI from 8-day datasets | NASA Land Processes Distributed Active Archive Center (LP DAAC), USGS/Earth Resources Observation and Science (EROS) Center, Sioux Falls, South Dakota,<https://lpdaac.usgs.gov/data_access/data_pool> |

Model validation

We conducted internal model validation using k-fold cross validation (Boyce et al. 2002). For mountain lions and wolves, we used 5-fold cross validation wherein the data sets were randomly partitioned into test and training sets. For each fold of withheld data, we generated predicted values from re-estimated model coefficients and used percentiles of predicted values in the available sample of withheld data to designate cut-off values among ordinal bins of habitat suitability, ranked lowest to highest from 1 to 5. We then validated models using a Spearman rank correlation test (*r_s_*) to compare the frequency of withheld used predator locations in each of 5 bins to each bin’s relative ranking. Finally, for the wolf resource selection models we conducted external model validation by testing the predictive performance of the top seasonal RSF models using the withheld VHF telemetry data that was not used in development of the RSF model. Furthermore, to assess how well the estimated, population-level models of resource selection for wolves and mountain lions could predict across individuals and years (for wolves) or across individuals (for mountain lions), we used leave-one-out cross validation of the RSF models. For each species, we iteratively fit a model to all the GPS data leaving out one individual, and then predicted selection patterns for the left-out individual with the estimated model.

**Results**

For wolves, we used a total of 1746 summer locations from the 4 individuals (average = 436.5 locations per individual, range = 60 to 1117 locations). We obtained a total of 160 summer VHF locations from 31 wolves (average = 10.3 locations per individual, range = 1 to 25). For mountain lions, we used a total of 7524 locations from the 13 individuals (average = 578.8 locations per individual, range = 514 to 653 locations). Both models internally validated very well based on the Spearman’s rank correlation test (mountain lion: Spearman’s rho = 0.998, p < 0.001; wolf model: Spearman’s rho = 1, p < 0.001). The wolf model validated well against the VHF data not used in the resource selection function estimation (Spearman’s rho = 0.912, p < 0.001). Finally, the models did an adequate job predicting patterns of use across individuals using leave-one-out cross-validation (wolves: Spearman’s rho = 1, p < 0.001, mountain lions: Spearman’s rho = 1, p < 0.001), which we took as support for our use of predicted relative probabilities of use for these predators as covariates for elk resource selection even if the time windows of location data did not completely align.

Wolf resource selection was a strong function of physiographic covariates (Figure A2). Wolves selected for intermediate elevations at relatively flat areas (i.e., low values of slope and slope variance), and close to water. Furthermore, we found strong evidence for an interaction such that selection for distance away from developed areas continued to increase in open areas (non-forested), whereas in forested areas selection initially increased to a plateau after which increased distance from development had less influence on selection. Finally, selection increased in association with higher values of NDVI.


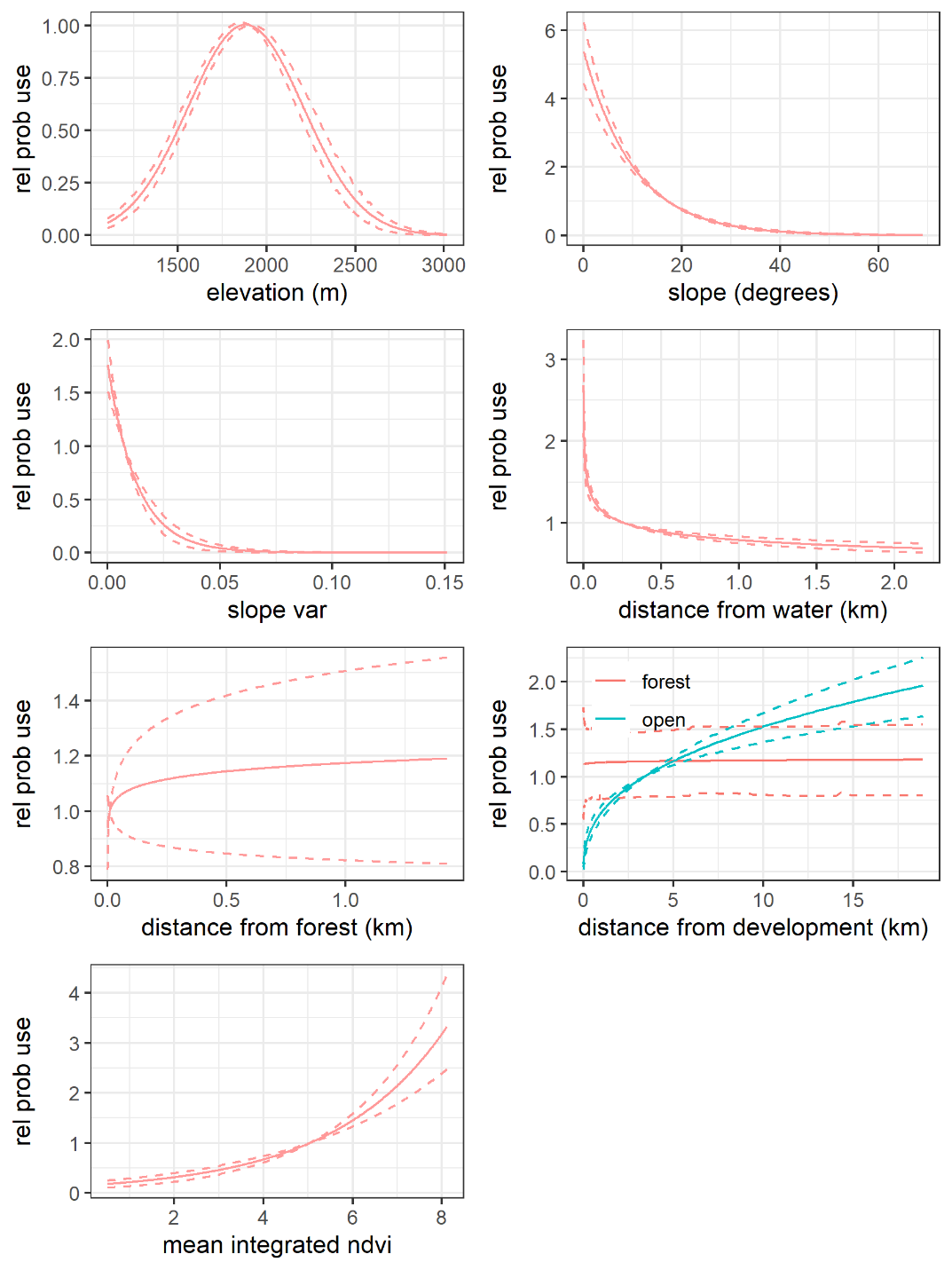


Figure A2. Predicted results from the top model for wolf resource selection. For each panel, predictions were made by holding other covariates to their mean values (with standardized covariates, a value of zero). The solid line indicates the median, and the dotted lines the 90% credible interval. The covariates have been back-transformed to their original scale.

Mountain lion resource selection was also a strong function of physiographic, development and environmental covariates (Figure A3). Mountain lions selected for low elevation areas with more rugged terrain (i.e., intermediate values of slope and high values of slope variance). Selection increased for areas further from forest and closer to water. Selection increased for areas further from development and motorized roads, although an interaction between landcover type (forested or not) and these two distance metrics suggested selection depended on whether mountain lions were in forested areas, e.g., selection actually decreased with distance from development if mountain lions were in forested areas. Finally, similar to wolves, selection increased for areas with higher values of integrated NDVI.


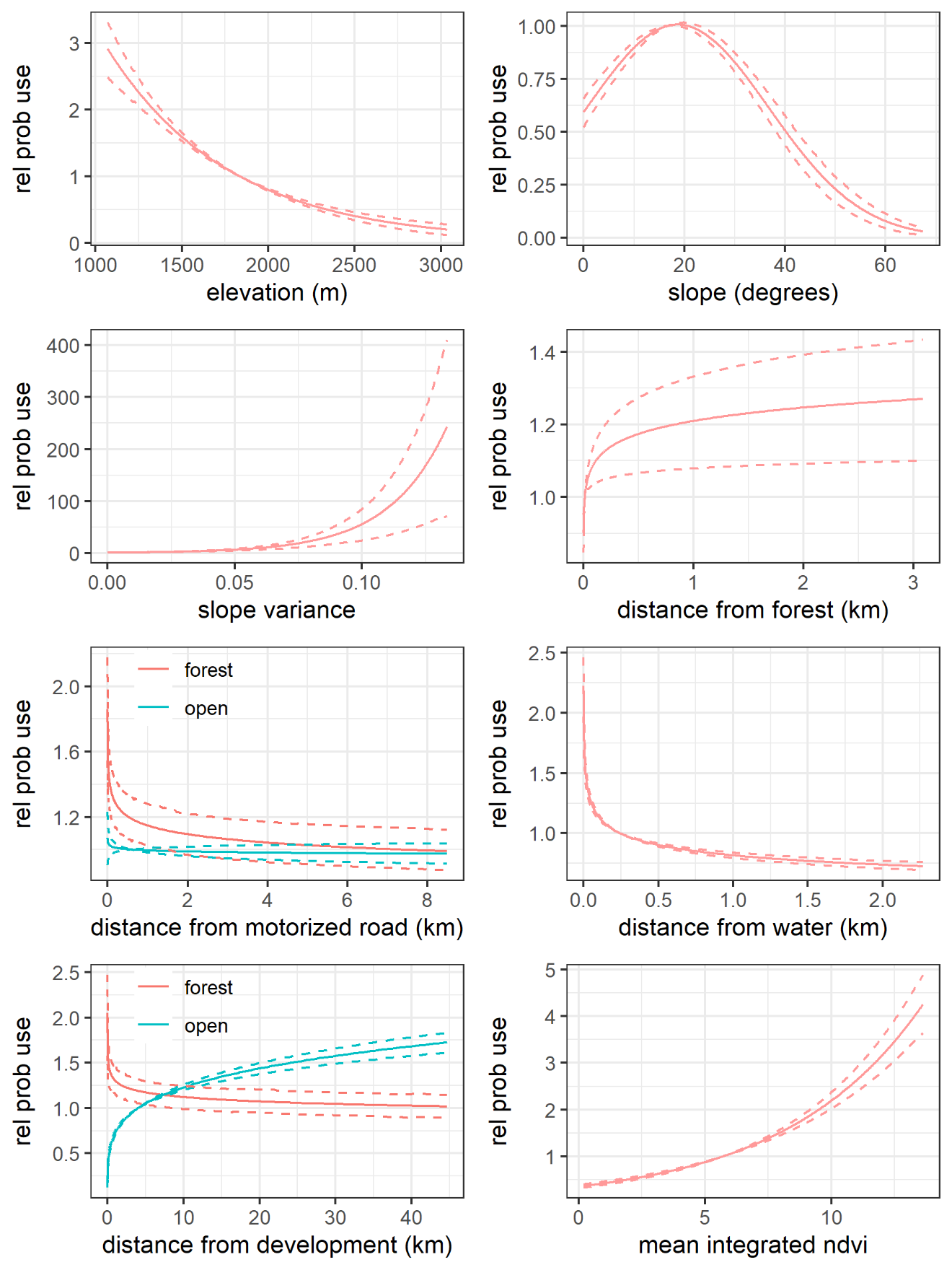


Figure A3. Predicted results from the top model for mountain lion resource selection. For each panel, predictions were made by holding other covariates to their mean values (with standardized covariates, a value of zero). The solid line indicates the median, and the dotted lines the 90% credible interval. The covariates have been back-transformed to their original scale.

**References**

Benson JF, Sikich JA, Riley SPD. 2016. Individual and population level resource selection patterns of mountain lions preying on mule deer along an urban-wildland Gradient. PLoS ONE 11: e0158006. https://doi.org/10.1371/journal.pone.0158006

Bergman, E. J. et al. 2006. Assessment of prey vulnerability through analysis of wolf movements and kill sites. - Ecol. Appl. 16: 273–284.

Blake, L. W. and Gese, E. M. 2016. Resource selection by cougars: Influence of behavioral state and season. - J. Wildl. Manag. 80: 1205–1217.

Boyce, M. S. et al. 2002. Evaluating resource selection functions. - Ecol. Model. 157: 281–300.

Burnham, K. P. et al. 2011. AIC model selection and multimodel inference in behavioral ecology: some background, observations, and comparisons. - Behav. Ecol. Sociobiol. 65: 23–35.

Calenge, C. 2006. The package adehabitat for the R software: tool for the analysis of space and habitat use by animals. - Ecol. Modelling 197: 516-519.

Fieberg, J., Matthiopoulous, J., Hebblewhite, M., Boyce, M.S., and Frair, J.L. 2009. Regression modelling of correlated data in ecology: subject-specific and population averaged response patterns. – Phil. Trans. Royal Soc. B: Biol. Sci 365: 2233-2244.

Golding, N. 2019. greta: Simple and scalable statistical modelling in R.

Hebblewhite, M. and Merrill, E. H. 2007. Multiscale wolf predation risk for elk: does migration reduce risk? - Oecologia 152: 377–387.

Hebblewhite, M. and Merrill, E. 2008. Modelling wildlife–human relationships for social species with mixed-effects resource selection models. - J. Appl. Ecol. 45: 834–844.

Johnson, D. H. 1980. The comparison of usage and availability measurements for evaluating resource preference. - Ecology 61: 65–71.

Johnson, C. J. et al. 2006. Resource selection functions based on use-availability data: theoretical motivation and evaluation methods. - J. Wildl. Manag. 70: 347–357.

Johnson, R.D., 2017. Mountain lion (*Puma concolor*) population characteristics and resource

selection in the North Dakota Badlands. - Thesis: South Dakota State University.

Kauffman, M. J. et al. 2007. Landscape heterogeneity shapes predation in a newly restored predator–prey system. - Ecol. Lett. 10: 690–700.

Manly, B. F. L. et al. 2007. Resource selection by animals: statistical design and analysis for field studies. - Springer Science & Business Media.

Pettorelli, N. et al. 2011. The Normalized Difference Vegetation Index (NDVI): unforeseen successes in animal ecology. - Clim. Res. 46: 15–27.

R Core Team 2018. R: a language and environment for statistical computing. - R Foundation for Statistical Computing.

Robinson, H.S., Ruth, T., Gude, J.A., Choate, D., DeSimone, R., Hebblewhite, M., Kunkel, K.,

Matchett, M.R., Mitchell, M.S., Murphy, K. and Williams, J., 2015. Linking resource selection and mortality modeling for population estimation of mountain lions in Montana. – Ecol. Mod. 312: 11-25.

Uboni, A., Smith, D.W., Mao, J.S., Stahler, D.R. and Vucetich, J.A., 2015. Long‐and short‐term

temporal variability in habitat selection of a top predator. Ecosphere 6: 1-16.

Appendix S2. Daily spatial patterns of elk.

Prior work has demonstrated that inference on spatial patterns of use by elk during the summer differ between datasets that are built on the full 24-hour day and those built using temporal subsets of the day (e.g., morning hours) (Beyer and Haufler 1994, Roberts et al. 2017). This is broadly consistent with the large body of evidence suggesting considerable variation in daily patterns of elk activity, e.g. foraging during dawn and dusk (Green and Bear 1990), selecting habitat to facilitate thermoregulation (Merrill 1991), and in response to peak activity periods of predators (Kohl et al. 2018). To investigate the influence of these daily patterns on our results, we adopted a two-pronged approach. First, we evaluated the strength of evidence for such variation in daily spatial use patterns by estimating the hourly mean step length (average distance between used points each hour) to identify potential temporal windows with disparate activity. Second, we estimated resource selection functions for each temporal window, and compared inference on population-level selection coefficients to evaluate how robust inference was.

We used the amt package (Signer et al. 2019) in the R programming environment (R Core Team 2018) to create a track for each individual GPS record and resample the record to 30-minute intervals. For each individual, we then calculated the mean step length between estimated locations for each hour of the day (Figure B1).


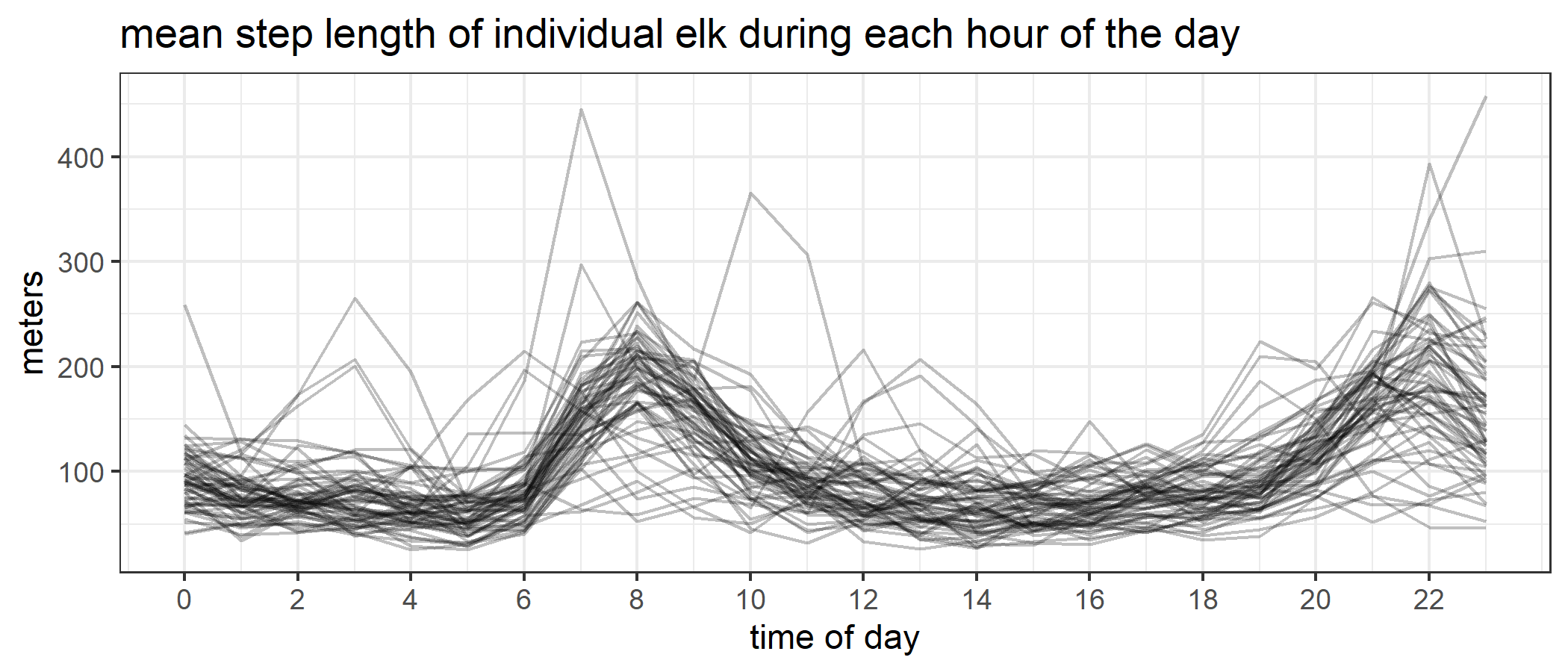


Figure B1. Mean step length between estimated locations of elk during hour of the day. This represents the average over the summer period of the distance moved during each hour.

Figure B1 reveals population-level periods of peak activity from 06:00 to 11:00, and from 18:00 to 24:00, and periods of minimal movement from 11:00 to 18:00 and 24:00 to 06:00. Periods of peak activity are consistent with elk feeding during dawn and dusk, the period from 24:00 to 06:00 is consistent with elk bedded down for the evening, and the period from 11:00 to 18:00 is consistent with inactivity in response to thermoregulation. Correspondingly, we identified four temporal windows: 1) the entire 24-hour period (FULL), 2) periods of peak activity (06:00 to 11:00 and 18:00 to 24:00, PEAK), 3) the 24-hour record excluding the period during the heat of the afternoon (11:00 to 18:00, EXC), and 4) the period of relative inactivity (11:00 to 18:00, HOT).

We built a separate dataset for each temporal period to use in the third-order resource selection analysis. Using the same procedures outlined in our Methods section, we then estimated selection coefficients for each period (Figure B2). The comparison strongly suggests that inference on selection is similar throughout the day, with the notable exception of selection for digestible energy (DE) during the hottest part of the day (11:00 to 18:00).


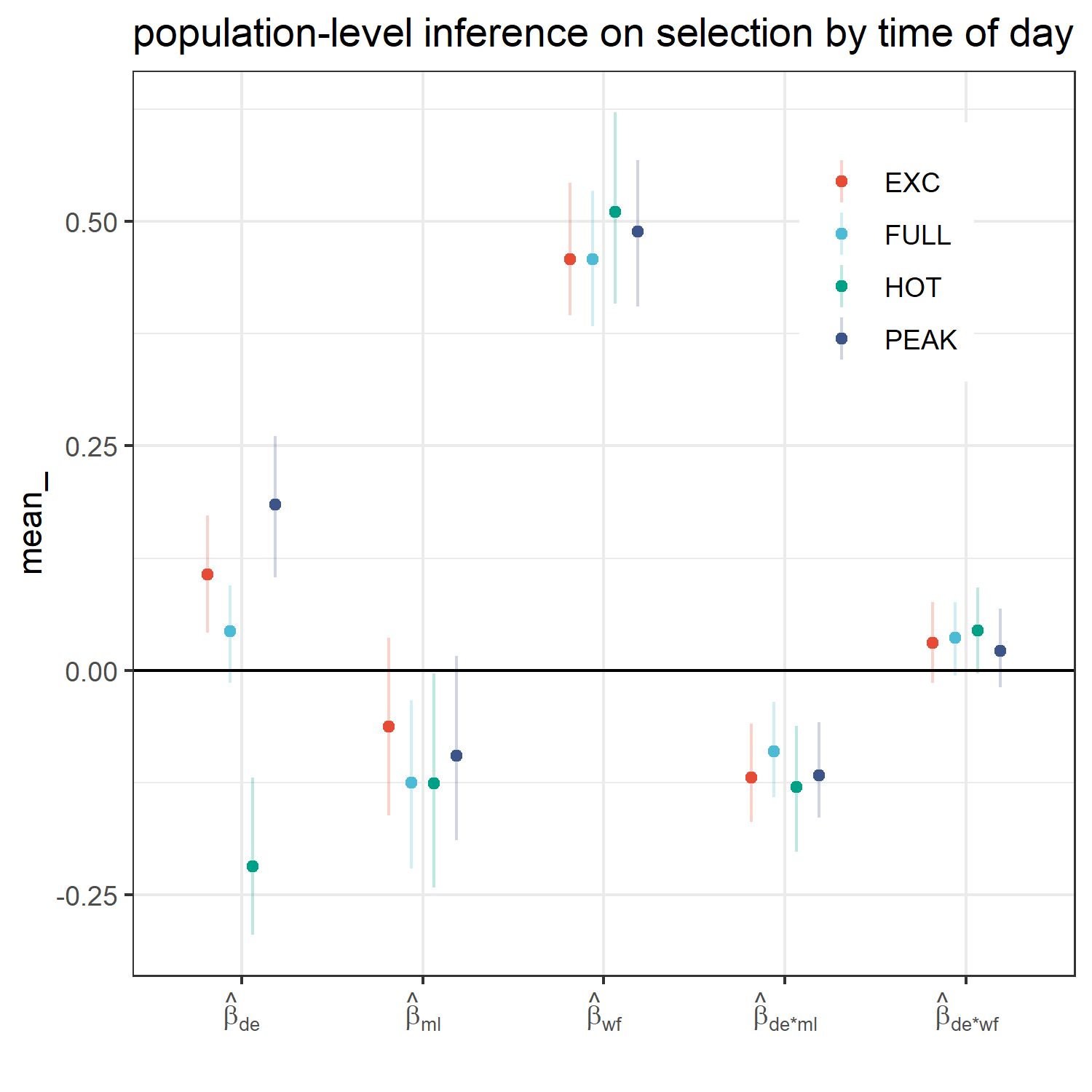


Figure B2. Estimated regression coefficients for four temporal windows.

Appendix S3. The landscapes of forage quality and risk.

A prerequisite for a trade-off between selection for forage quality and risk is a correlation between the covariates on the landscape, i.e., a trade-off cannot exist where an individual is never faced with a choice between high quality forage and higher risk. Using the used-available data sets, we found that there was a significant correlation between forage quality (as indexed by digestible energy) and both wolf and mountain lion risk (indexed by predicted relative probabilities of selection) at both the second and third orders of resource selection (Figure C1).


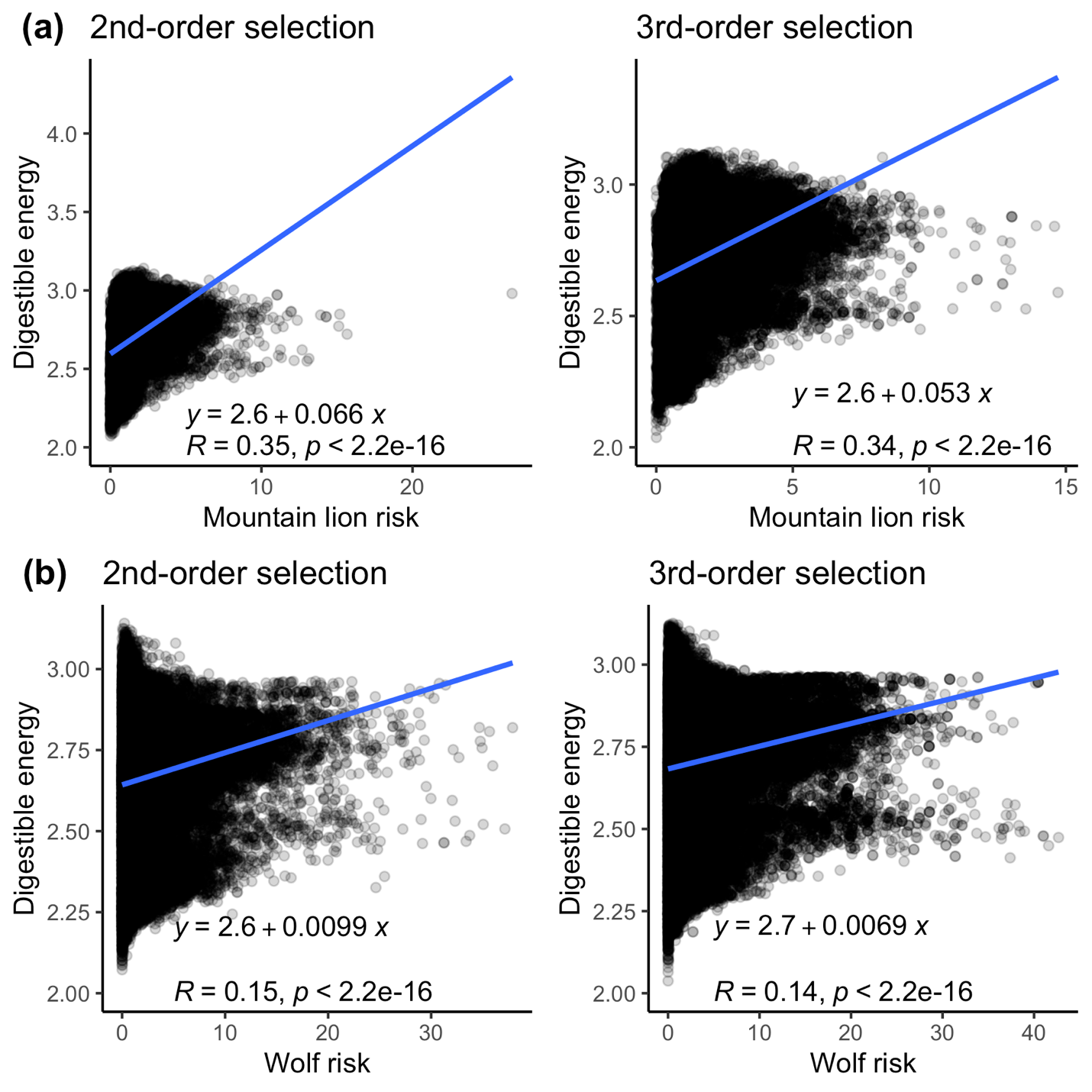


Figure C1. Correlation between forage quality and risk (mountain lions, panel a, wolves, panel b) at both orders of resource selection by elk.
